# An alternative cytoplasmic SFPQ isoform with reduced phase separation potential is up-regulated in ALS

**DOI:** 10.1126/sciadv.adt4814

**Published:** 2025-08-22

**Authors:** Jacob Neeves, Marija Petrić Howe, Oliver J. Ziff, Beth Callaghan, Daniel Jutzi, Koustav Pal, Theodoros I. Roumeliotis, Jyoti Choudhary, Adrian M. Isaacs, Frank Rigo, C. Frank Bennett, Marc-David Ruepp, Rickie Patani

**Affiliations:** ^1^ Department of Neuromuscular Diseases, Queen Square Institute of Neurology, University College London, London WC1N 3BG, UK.; ^2^ The Francis Crick Institute, 1 Midland Road, London NW1 1AT, UK.; ^3^ UK Dementia Research Institute at King’s College London, London, UK.; ^4^ Department of Basic and Clinical Neuroscience, Institute of Psychiatry, Psychology & Neuroscience, King’s College London, 125 Coldharbour Lane, London SE5 9NU, UK.; ^5^ Functional Proteomics team, The Institute of Cancer Research, 237 Fulham Road, London SW3 6JB, UK.; ^6^ UK Dementia Research Institute at University College London, London, UK.; ^7^ Department of Neurodegenerative Disease, Queen Square Institute of Neurology, University College London, London WC1N 3BG, UK.; ^8^ Ionis Pharmaceuticals, Carlsbad, CA 92010, USA.

## Abstract

Splicing factor proline- and glutamine-rich (SFPQ) is an RNA binding protein that broadly regulates RNA metabolism. Although its nuclear roles are well studied, evidence of SFPQ’s cytoplasmic functionality is emerging. Altered expression and nuclear-to-cytoplasmic redistribution of SFPQ have been recognized in amyotrophic lateral sclerosis (ALS) pathology, yet the mechanistic bases for these phenomena remain undetermined. We identified altered *SFPQ* splicing in ALS, increasing the expression of an alternative mRNA isoform lacking a nuclear localization sequence, which we termed “*altSFPQ*.” We find that *altSFPQ* mRNA contributes to SFPQ autoregulation and is highly unstable yet exhibits context-specific translation with cytoplasm-predominant localization. Notably, reduced canonical *SFPQ* coincides with increased *altSFPQ* transcript expression in familial and sporadic ALS models, providing a mechanistic basis for SFPQ nuclear-to-cytoplasmic redistribution in patients with ALS. Last, we observe that the altSFPQ protein has reduced phase separation potential and differential protein binding compared to its canonical counterpart, providing insight into its mechanistic relevance to physiology and ALS pathogenesis.

## Teaser

Mechanisms driving nucleocytoplasmic distribution of SFPQ proteins in health and ALS are uncovered.

## INTRODUCTION

Amyotrophic lateral sclerosis (ALS) is a devastating disease with a lifetime risk of between 1:300 and 1:400. It is among the most rapidly progressive and relentless neurodegenerative diseases, with an average survival of only 3 to 5 years. The need for effective disease-modifying treatment targeting the full spectrum of ALS is apparent and urgent. However, a prerequisite for achieving this is a deeper understanding of its molecular pathomechanisms. Recognized pathological hallmarks of ALS include the nuclear-to-cytoplasmic redistribution of RNA binding proteins (RBPs), including TAR DNA binding protein of 43 kDa (TDP-43), fused in sarcoma (FUS), and splicing factor proline- and glutamine-rich (SFPQ) (*1*–*5*). Autosomal-dominant mutations are prevalent within the nuclear localization signal (NLS) region in *FUS*-ALS and less common, but reported, in *TARDBP*-ALS. Beyond these cases, the mechanistic basis for RBP redistribution in ALS, including for SFPQ, is incompletely resolved.

SFPQ is an abundant, nuclear-predominant RBP, involved in many aspects of nuclear RNA metabolism (*6*), including transcriptional regulation (*7*), pre-mRNA splicing (*8*), and paraspeckle formation (*9*). Consequently, its dysregulated expression and localization cause widespread dysfunction, particularly in neurons, which is typically attributed to loss of its nuclear functions. Loss of SFPQ results in the inclusion of numerous cryptic last exons in genes with neuronal functions (*10*), as well as neuronal apoptosis due to reduced RNA polymerase II processivity through long genes (*11*). Furthermore, spatial dissociation of nuclear SFPQ and FUS, which normally form a high–molecular weight (HMW) complex, causes increased four-repeat tau isoform expression, which, in turn, leads to frontotemporal dementia (FTD)–like phenotypes in mice (*12*) and neurodegeneration in human cortical neurons (*13*). In addition to its nuclear functions, cytoplasmic roles of SFPQ, particularly in neurons, have also been described (*14*–*16*). Notably, SFPQ knockout primarily affects neuronal development, manifesting in axonal malformation, which is abrogated through expression of a cytoplasmically restricted SFPQ protein (ΔNLS-wtSFPQ) (*17*). This demonstrates the crucial role played by a non-nuclear pool of SFPQ in neurons.

Pathophysiological increases in cytoplasmic SFPQ, including aggregate formation, have been described in several neurodegenerative diseases (*5*, *18*–*21*). However, the effect of these phenomena on affected cell types and the mechanistic basis for altered localization remains undetermined. Recently, *SFPQ* gene polymorphisms were identified in patients with ALS, found in the coiled-coil region implicated in zinc-mediated subcellular localization (*19*, *22*). Considering that these regions of the protein are shared with the other two members of the *Drosophila* behavior/human splicing (DBHS) proteins, PSPC1 and NONO, which do not exhibit significant redistribution in ALS (*23*), it follows that alternative regulation may drive cytoplasmic localization of SFPQ. Against this background, we hypothesized that an alternative splicing event determines SFPQ nucleocytoplasmic distribution. Along with protein dysregulation, we and others recently described aberrant alternative splicing of *SFPQ*, in the form of intron retention (IR), in multiple ALS subtypes (*5*, *24*, *25*). Although *SFPQ* transcript isoforms have previously been described (*26*), overall regulation of *SFPQ* pre-mRNA splicing and the roles of its RNA products remain unresolved.

In this study, we show that an alternative distal exon event in *SFPQ* is expressed throughout motor neuron development, anticorrelates with the major coding mRNA, and is highly unstable due to nonsense-mediated mRNA decay (NMD). This mRNA also exhibits translation potential and produces a cytoplasmic-predominant protein with differential phase separation propensity and protein binding partners compared to the canonical SFPQ protein. This RNA isoform is up-regulated in human induced pluripotent stem cell (hiPSC)–derived familial ALS neurons (*VCP*-, *FUS*-, and *SOD1*- mutations) and in motor neurons derived from patients with sporadic ALS, thus representing the vast majority of ALS cases. Therefore, we propose that aberrant regulation of *SFPQ* gene expression ultimately contributes to driving its nuclear protein loss and cytoplasmic accumulation through the preferential production of a cytoplasmically localized protein.

## RESULTS

### *SFPQ* alternative distal exon splicing is ubiquitous and well conserved

**Fig. 1.**
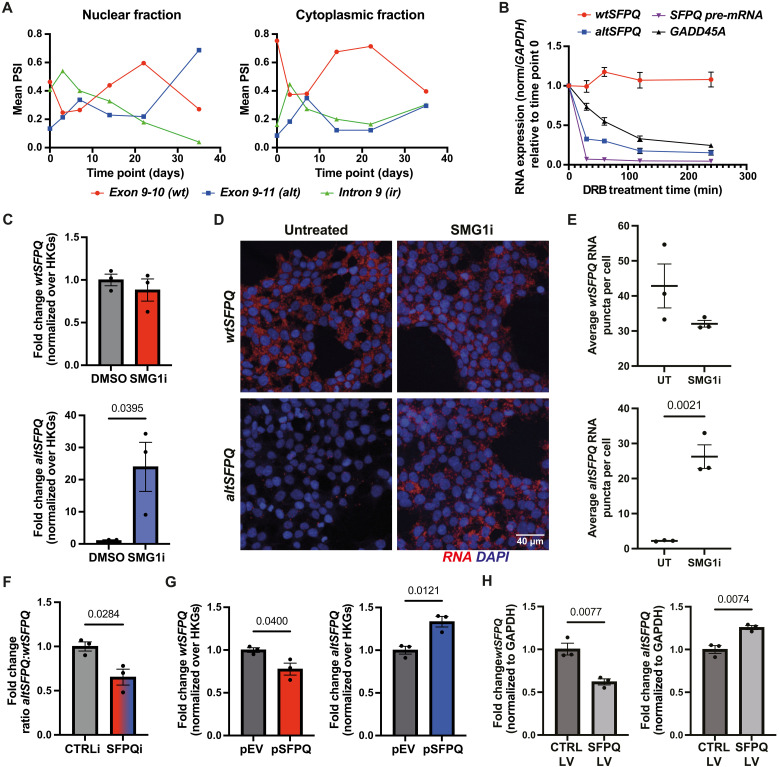
*AltSFPQ* is an NMD target and contributes to SFPQ autoregulation. (**A**) Mean percentage splicing (PSI) for each of the three splicing events across six stages of neuronal differentiation (see fig. S1C) in either nuclear or cytoplasmic fractions derived from four healthy hiPSC lines. (**B**) RNA expression (qPCR) relative to time point 0 when DRB treatment was administered, normalized at each time point over *GAPDH*, in HEK293T cells (*n* = 3). (**C**) *WtSFPQ* and *altSFPQ* mRNA levels (qPCR) normalized over *GAPDH* and *POLR2B*, in SMG1 inhibitor (SMG1i) and mock-treated HEK293T cells (*n* = 3; unpaired *t* tests). (**D**) Representative images of BaseScope RNA-FISH on untreated and SMG1i-treated HEK293T cells, probed for either *wtSFPQ* or *altSFPQ*. (**E**) Quantification of BaseScope *SFPQ* RNA puncta per cell in untreated (UT) and SMG1i-treated HEK293T cells (*n* = 3; average of ≥4 fields per replicate; unpaired *t* tests). (**F**) Ratio between *altSFPQ* and *wtSFPQ* transcript expression as measured by qPCR in SFPQ siRNA-treated hiPSC-derived DIV3 motor neurons (*n* = 3; unpaired *t* test). (**G**) *WtSFPQ* and *altSFPQ* mRNA expression levels (qPCR) normalized over *GAPDH* and *POLR2B*, in the HA-wtSFPQ plasmid (pSFPQ) and control plasmid (pEV) transfected HeLa cells (*n* = 3; unpaired *t* tests). (**H**) *WtSFPQ* and *altSFPQ* mRNA expression levels (qPCR) normalized over *GAPDH*, in wtSFPQ-T2A-mAPPLE and mAPPLE control lentiviral transduced DIV14 (day 11 posttransduction) i3Neurons (*n* = 3; unpaired *t* tests). Graphs are presented as means ± SEM. HKGs, housekeeping genes.

To gain initial insight into *SFPQ* gene expression, we searched the UCSC genome browser for annotated *SFPQ* transcripts. The NCBI RefSeq collection includes the major protein-coding mRNA and two noncoding transcripts, which use alternative terminal exons; these two transcripts differ by just three nucleotides (fig. S1A, in blue). Whereas the first alternative distal exon is well conserved, two additional downstream exons have arisen in mammals. Ensembl includes at least 10 annotated human transcripts; ENST00000357214 is denoted as coding, all others as noncoding (fig. S1A, in red). To broadly assess the relative expression of these transcripts, we used the ExonSkipDB web browser focusing on the Genotype-Tissue Expression (GTEx) database of RNA sequencing (RNA-seq) across 31 normal human tissues [https://ccsm.uth.edu/ExonSkipDB/; (*27*)] (fig. S1B). This demonstrated that three transcripts are ubiquitously expressed: the only annotated coding transcript (henceforth termed *wtSFPQ*, ENST00000357214), an alternative distal exon(s) transcript (henceforth termed *altSFPQ*, ENST00000470472), and an additional noncoding transcript consistent with the intron-retaining RNA we previously identified [ENST00000468598; (*5*)]. ENST00000470472 corresponds to RefSeq NR_136703.2, whereas the lowly expressed ENST00000460428 corresponds to RefSeq NR_136702.2. The three transcripts are expressed in all tissues, with relatively high expression in nerve tissue (fig. S1B). Last, we performed MAJIQ splicing analysis (*28*, *29*) on our previously generated RNA-seq data derived from samples taken throughout directed motor neurogenesis from hiPSCs (six stages in total, including two postmitotic neuronal stages), with both nuclear and cytoplasmic fractions analyzed at each stage (fig. S1C). MAJIQ quantifies both annotated and unannotated splicing events. The local splicing variant (LSV) which used multiple well-expressed events in both cell compartments and across all cell stages was chr1:35187001-35187122. This LSV diverges from exon 9 and contains (i) exon 9 to 10 splicing, consistent with canonical protein-coding *wtSFPQ*; (ii) exon 9 to 11 splicing, consistent with *altSFPQ*; and (iii) previously identified intron 9 retention (*5*) (a simplified SFPQ schema demonstrating the divergent events described, and primer design to specifically assay them, is shown in fig. S1D). Each isoform is detected in both cell compartments throughout motor neurogenesis, but the relative expression varies in a stage-dependent manner. An anticorrelation was observed with respect to the relative levels of exon 9 to 10 (*wt*) and exon 9 to 11 (*alt*) splicing in both compartments. Intron 9 inclusion (*ir*) also exhibits an anticorrelation with exon 9 to 10 splicing at most stages, particularly within cytoplasmic samples (Fig. 1A). Therefore, our data suggest that these splicing events in *SFPQ* are ubiquitous across tissues and expressed continuously through neurodevelopmental lineage restriction of motor neurons. Notably, relative to *wtSFPQ* expression, absolute levels of *altSFPQ*, as determined by reverse transcription quantitative polymerase chain reaction (RT-qPCR), increase through motor neurogenesis from iPSCs, to DIV14 neural precursor cells (NPCs), then to DIV35 terminally differentiated motor neurons (fig. S2A). Nevertheless, relatively low expression of *altSFPQ* across tissues and at earlier stages of our neurogenesis paradigm implies a lower nascent transcription rate and/or reduced posttranscriptional stability compared to *wtSFPQ*.

### *AltSFPQ* is an NMD target and contributes to SFPQ autoregulation

To infer posttranscriptional stability of the *altSFPQ* transcript, we treated human embryonic kidney (HEK) 293T cells with the transcriptional inhibitor 5,6-dichloro-1-β-ribofuranosyl benzimidazole (DRB) and assessed RNA expression over time by RT-qPCR. Compared to the relatively stable transcript *GAPDH*, *altSFPQ* was highly unstable, exhibiting down-regulation almost comparable to an *SFPQ* constitutively spliced intron and at a faster rate than the established NMD-sensitive target *GADD45A* (Fig. 1B). We orthogonally validated this finding by using the highly selective RNA polymerase II irreversible inhibitor alpha-amanitin (*30*, *31*) (fig. S2B). When considered together with the cytoplasmic presence of *altSFPQ* mRNA, cytoplasmic degradation is implicated. *AltSFPQ* contains a premature translation termination codon (PTC), followed by two downstream introns, which suggests that it is a target for NMD (*32*, *33*). The *SFPQ* intron-retaining transcript also introduces PTCs and is present in the cytoplasm (*5*, *25*). To evaluate the NMD sensitivity of these transcripts, HEK293T cells were treated with the mRNA translation inhibitor cycloheximide (CHX). *AltSFPQ* mRNA exhibited a marked 20-fold up-regulation upon inhibition of translation (fig. S2C), which was threefold greater than *GADD45A*. Intron-retaining *SFPQ* (henceforth referred to as *irSFPQ*) exhibited no change in expression, whereas *wtSFPQ*, unexpectedly, exhibited a small but significant up-regulation. To confirm that exon 9 to 11 splicing reflects the full-length *altSFPQ* transcript, we performed Northern blot analysis on CHX-treated HEK293T RNA. Using a probe designed to span the last three exons of *altSFPQ*, we demonstrated clear up-regulation of a 2.3-kb transcript consistent with *altSFPQ* transcript size (fig. S2D). To orthogonally validate that *altSFPQ*, but not *wtSFPQ*, is an authentic NMD target (*34*), we used a chemical inhibitor of SMG1 kinase, a crucial component of the NMD pathway (*35*). Following a 24-hour treatment, *altSFPQ* transcript levels rose markedly in HEK293T cells as measured by RT-qPCR, whereas *wtSFPQ* was unaltered (Fig. 1C). This was further orthogonally demonstrated by BaseScope RNA-FISH on fixed HEK293T cultures. Average *altSFPQ* RNA puncta per cell rose from 2.23 to 26.24 in SMG1i treatment, whereas *wtSFPQ* RNA levels nonsignificantly decreased from 42.86 to 32.06 per cell (Fig. 1, D and E). Recognizing cell type–specific differences in NMD activity, we further confirmed these findings in hiPSC-derived motor neurons treated with the SMG1 inhibitor. In this context, this finding was coupled with a significant reduction in *wtSFPQ* expression. Lack of *GADD45A* response in neurons, but stabilization in HEK293T cells may reflect a reduced concentration of the inhibitor used in neurons or cell type–specific differences in NMD activity (fig. S2, E and F). Nonetheless, together, these data strongly suggest that *altSFPQ* is an authentic NMD target, but *irSFPQ* is not.

Many RBPs autoregulate (*36*–*38*). Noting that (i) the SFPQ protein binds extensively to its own transcripts (*5*) and (ii) *altSFPQ* transcript is highly unstable, we hypothesized that SFPQ protein autoregulates by directly modulating the splicing decision between *altSFPQ* and *wtSFPQ*. To address this, we performed small interfering RNA (siRNA)–mediated knockdown of SFPQ protein in hiPSC-derived motor neurons. We found that, although both *wtSFPQ* and *altSFPQ* transcripts were substantially down-regulated, there was a greater magnitude of reduction in *altSFPQ* (Fig. 1F and fig. S2G). These changes occurred in the absence of altered *SFPQ pre-mRNA* levels, as inferred by measuring expression of a constitutively spliced intron. We next investigated the effects of SFPQ overexpression on endogenous *SFPQ* RNAs via transient transfection of a hemagglutinin (HA)–tagged wtSFPQ construct in HeLa cells (fig. S2H). We observed a significant down-regulation of endogenous *wtSFPQ* transcript 48 hours posttransfection along with significantly increased *altSFPQ* but no change in *irSFPQ* or *pre-mRNA* (Fig. 1G and fig. S2I). Last, we overexpressed HA-wtSFPQ in i3Neurons via lentiviral transduction; again, *wtSFPQ* was significantly down-regulated and *altSFPQ* up-regulated (Fig. 1H). Together, these data support a model in which SFPQ protein autoregulates through favored splicing of an mRNA which is targeted for degradation via NMD.

### *AltSFPQ* encodes a previously unidentified SFPQ protein

**Fig. 2.**
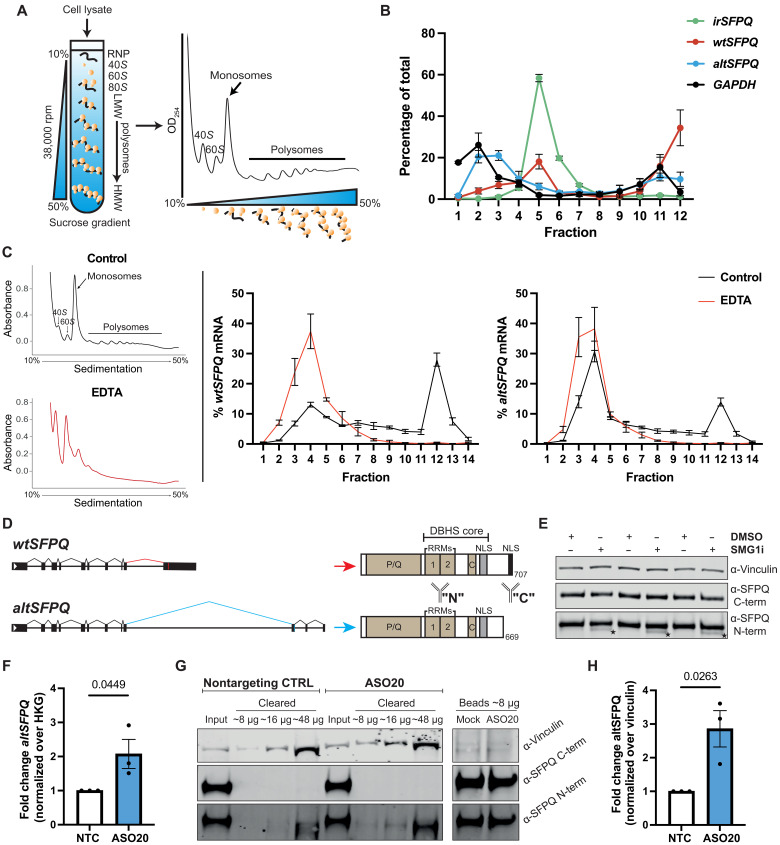
*AltSFPQ* encodes a previously unidentified SFPQ protein. (**A**) Polysome profiling schema, with example absorbance profile. RNP, ribonucleoprotein. (**B**) Analysis of *SFPQ* transcripts and *GAPDH* mRNA by qPCR from hiPSC-derived NPCs (DIV7), plotted as the percentage of total (*n* = 3; one cell line derived from a healthy donor and two cell lines derived from a patient with VCP-ALS). (**C**) Polysome profiles obtained from hiPSC-derived DIV14 NPCs with (red) or without (black) EDTA treatment; EDTA treatment caused substantial loss of polysomes. Analysis (right) of *wtSFPQ* or *altSFPQ* mRNA expression by qPCR from each fraction, plotted as the percentage of total (*n* = 3; two cell lines from healthy donors and one cell line from a patient with VCP-ALS). (**D**) Schematic depiction of SFPQ isoforms and antibodies used for IP and immunoblotting. One antibody recognizes an epitope common to both proteins (“N”-term SFPQ antibody), whereas a C-terminally targeting antibody recognizes only wtSFPQ (“C”-term SFPQ antibody). RRMs, RNA recognition motifs. (**E**) Western blot showing the altSFPQ isoform expression in SMG1i-treated HEK293T cells; *n* = 3, stars demarcate the proteins. (**F**) *AltSFPQ* mRNA levels (qPCR, normalized over *GAPDH*) in nontargeting control ASO (NTC)–treated and ASO20-treated HEK293T cells (*n* = 3; unpaired *t* test). (**G**) Representative Western blot of the endogenous SFPQ protein expression in NTC-treated or ASO20-treated HEK293T cells; relates to (F). Initial IP using ab177149 (C terminus targeting) SFPQ antibody removes wtSFPQ and enables visualization of the altSFPQ protein in “cleared” fractions (using N-terminal SFPQ antibody). An 8-μg input was loaded along with increasing amounts of cleared lysate; an input equivalent amount of the bead eluate was loaded. (**H**) Quantification of altSFPQ (normalized over vinculin) in (G), using the highest-quantity cleared lysate within each replicate (*n* = 3; unpaired *t* test). Graphs are presented as means ± SEM.

Lack of NMD sensitivity of *irSFPQ* and partial cytoplasmic localization prompted us to examine its coding potential. To do so, we performed polysome profiling (Fig. 2A) on hiPSC-derived NPCs and subsequent RT-qPCR of transcripts on the resultant fractions. As expected, *wtSFPQ* and positive control *GAPDH* transcripts predominantly associate with HMW polysomal fractions, consistent with highly efficient translation. In contrast, *irSFPQ* was only localized to LMW polysome fractions. Considering that NMD is dependent on transcript engagement with ribosomal machinery, whereupon it is typically degraded during early rounds of translation, we also expected to see predominant *altSFPQ* engagement with individual ribosomes (monosomes) and low–molecular weight (LMW) polysomes but minimal engagement with HMW polysomes (*39*, *40*). Unexpectedly, a relatively large pool of *altSFPQ* mRNA associates with HMW polysomes, indicative of translation. The proportion of the transcript within these fractions is comparable to that of *GAPDH* (Fig. 2B). To ensure that these profiles reflected true ribosomal engagement and not cosedimentation, we first assessed the impact of EDTA treatment on HEK293T lysates to induce ribosomal dissociation. Both *wtSFPQ* and *altSFPQ* mRNAs shifted substantially from HMW polysome fractions toward lighter fractions (fig. S3A). The same effect was then observed following EDTA treatment of NPC lysates (Fig. 2C); notably, no shift was observed for *irSFPQ* (fig. S3B). To determine that *altSFPQ* polysomal association did not reflect a nonspecific or artifactual profile, we examined the *SRSF6* PTC+ NMD-sensitive transcript (*39*). The *SRSF6* transcript was predominantly identified in the monosomal fraction with progressively diminishing presence through disome and LMW polysome fractions. Furthermore, the different profile compared to *altSFPQ* is not simply a consequence of differential expression of the two transcripts (fig. S3C). Together, these data suggest *altSFPQ* translation potential in mammalian cells.

Specific identification of the alternative protein is complicated by (i) low expression in the basal state, (ii) similar molecular weight to the canonical SFPQ protein (wtSFPQ), and (iii) lack of tryptic digest sites to enable unique peptide identification, of relevance to most proteomics library preparation approaches. Commercially available antibodies include one that only targets the wtSFPQ C terminus and others that target structured domains common to both isoforms (hereafter denoted as N-terminal antibody) (Fig. 2D). HA-tagged wtSFPQ and altSFPQ proteins run at such similar weights as to overlap on SDS–polyacrylamide gel electrophoresis (PAGE) (fig. S3D). Consequently, and considering the relatively low expression of altSFPQ compared to wtSFPQ, higher input requirements will typically result in masking of the alternative protein. To circumvent this problem, we made use of SMG1 inhibition–mediated *altSFPQ* up-regulation in conjunction with long SDS-PAGE runtimes on fixed percentage gels and Western blot detection using both the N- and C-terminal antibodies. This demonstrated an α-N-terminal SFPQ-positive, α-C-terminal SFPQ-negative protein running at the expected molecular weight and therefore consistent with the novel isoform (Fig. 2E). To further confirm that this is an SFPQ protein, we pretreated cells with an SFPQ-targeting siRNA pool before SMG1i treatment. This reduced expression of the SMG1i-induced protein to less than 40% (fig. S3E), confirming that it is an SFPQ protein.

Reduced expression of NMD regulators and reduced UPF1 phosphorylation may result in broad translation of truncated proteins deriving from NMD-sensitive transcripts (*41*, *42*). To examine whether the altSFPQ protein is translated in the absence of NMD inhibition, we sought specific means of *altSFPQ* up-regulation. We reasoned that splicing of its encoding transcript might be determined by intronic splicing regulatory sequences in close proximity to the splice sites. Therefore, we designed a panel of uniformly phosphorothioate (PS)- and 2′-*O*-methoxyethyl (2′MOE)–modified antisense oligonucleotides (ASOs) with the aim of specifically increasing *altSFPQ* expression. In total, 20 ASOs were designed and tested: 2 ASOs targeting either the 5′ or 3′ splice site, with respect to *altSFPQ* exons 9 and 10, and a further 18 ASOs “tiled” in nine-nucleotide steps forward or backward from the 5′ and 3′ splice sites, respectively (fig. S3F). ASOs were initially screened in HEK293T cells for 48 hours at a 200 nM concentration, and *SFPQ* mRNA expression was assessed by RT-qPCR. One ASO (ASO20) successfully increased *altSFPQ* mRNA expression (fig. S3G). To maximize accuracy of altSFPQ protein detection, we made use of the wtSFPQ C-terminal–specific antibody to immunoprecipitate this protein out of solution. Following this, immunoblotting using an SFPQ N-terminal–specific antibody revealed a protein ~97 kDa in size, with increased expression in an ASO20-dependent manner (Fig. 2, F to H). Of note, ASO20 treatment also reduced the expression of the *wtSFPQ* mRNA and wtSFPQ protein (fig. S3, H and I). Last, we used the previously described C-terminal antibody immunoprecipitation (IP) approach to confirm that the altSFPQ protein is expressed in hiPSC-derived motor neurons (fig. S3J). Together, this shows that an altSFPQ protein is expressed and that its levels increase when *altSFPQ* transcript expression is up-regulated.

### *AltSFPQ* encodes a cytoplasm-predominant protein, which attenuates subcellular distribution of DBHS proteins

**Fig. 3.**
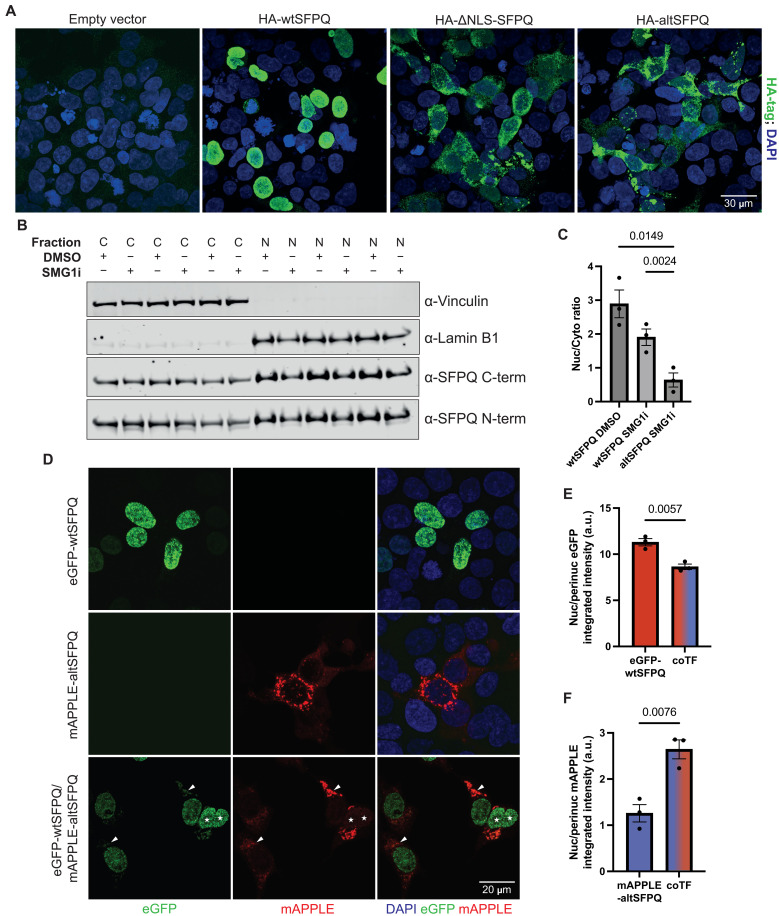
*AltSFPQ* encodes a cytoplasm-predominant protein, which attenuates subcellular distribution of DBHS proteins. (**A**) Representative immunofluorescence images showing the subcellular localization of HA-tagged recombinant SFPQ proteins in transfected HEK293T cells. (**B**) Western blot of nuclear-cytoplasmic fractionated SMG1i-treated HEK293T cells; vinculin and lamin B1 act as subcellular markers. (**C**) Quantification of the nuclear-cytoplasmic ratio of SFPQ proteins in fractionated SMG1i Western blots; the C-term SFPQ antibody signal is used to quantify wtSFPQ, and the N-term antibody is used to quantify the smaller visible SFPQ protein (i.e., altSFPQ); *n* = 3; one-way ANOVA, Tukey’s multiple comparisons. (**D**) HEK293T cells were transfected with 100 ng of eGFP-wtSFPQ (+100 ng of EV plasmid), 100 ng of mAPPLE-wtSFPQ (+100 ng of EV plasmid), or 100 ng of each tagged SFPQ protein; representative zoomed-in images are displayed (arrowheads show co-localized cytoplasmic eGFP and mAPPLE signals; stars show the nuclei exhibiting both eGFP and mAPPLE signal). (**E**) Image analysis quantification relating to (D), measuring the nuclear-cytoplasmic ratio (using a 20-μm perinuclear ring region) of the eGFP signal (reflecting the eGFP-wtSFPQ protein) in eGFP-wtSFPQ transfected versus eGFP-wtSFPQ/mAPPLE-altSFPQ cotransfected cells (*n* = 3; each data point represents the average across ≥5 fields of view for each replicate; unpaired *t* test). a.u., arbitrary units. (**F**) As for (E) but assessing the nuclear-cytoplasmic ratio of the mAPPLE signal (reflecting the mAPPLE-altSFPQ protein) (*n* = 3; each data point represents the average of ≥5 fields of view for each replicate; unpaired *t* test).

AltSFPQ and wtSFPQ proteins share an identical sequence until amino acid 663; altSFPQ has a further seven unique amino acids (total length of 669 amino acids), whereas wtSFPQ has an additional 45 unique amino acids (total length of 707 amino acids). In the altSFPQ protein, this difference dictates an omission of the classical nuclear localization signal (cNLS) located at the extreme C terminus of wtSFPQ (Fig. 2D), which is required for its nuclear localization (*43*). To test the localization of the altSFPQ protein, we transiently expressed HA-tagged wtSFPQ, altSFPQ, and a ΔNLS SFPQ (but otherwise unaltered from wtSFPQ) in HEK293T cells and assessed distribution by immunofluorescence (Fig. 3A). As expected, wtSFPQ was predominantly localized in the nucleus, whereas omission of the NLS markedly shifts the protein into the cytoplasm, although some nuclear localization remains. The altSFPQ protein exhibited a highly similar subcellular distribution as for the ΔNLS protein, demonstrating that the unique C terminus does not encode an alternative functional NLS. To assess the nuclear-cytoplasmic distribution of endogenous wtSFPQ and altSFPQ proteins, we used SMG1i treatment of HEK293T cells followed by biochemical fractionation and Western blotting. In line with the distribution of the recombinant proteins, wtSFPQ is predominantly nuclear, whereas SMG1i-induced altSFPQ is predominantly cytoplasmic, although both are present within both compartments (Fig. 3, B and C). To address whether altSFPQ can modulate *SFPQ* expression in a similar manner to wtSFPQ, we assessed the effect of altSFPQ overexpression on endogenous *SFPQ* RNAs. Transfected HA-altSFPQ did not significantly alter expression of *wtSFPQ* and *altSFPQ*, or *irSFPQ*, in HeLa cells (fig. S4A). Furthermore, lentiviral-derived HA-altSFPQ overexpression in i3Neurons also had no significant effect on the expression of *SFPQ* RNAs (fig. S4B). This implies that altSFPQ does not autoregulate.

The DBHS proteins (SFPQ, NONO, and PSPC1) primarily exist in homo- and heterodimeric forms (*44*). To determine whether altSFPQ expression modulates the subcellular distribution of wtSFPQ, we individually or coexpressed both with different fluorescent tags to visualize the two proteins simultaneously. Intriguingly, the recombinant proteins both affected one another’s localization, with redistribution of wtSFPQ to the cytoplasm and altSFPQ to the nucleus when coexpressed (Fig. 3, D to F). Furthermore, overexpressed altSFPQ, as well as a cytoplasmic wtSFPQ protein (ΔNLS-wtSFPQ), redistributed endogenous NONO, the highest-affinity heterodimeric partner of SFPQ protein (*45*), into the cytoplasm (fig. S4, C and D). Therefore, *altSFPQ* encodes a cytoplasmic protein, which can modulate the subcellular distribution of other DBHS proteins, likely through dimerization.

### AltSFPQ exhibits reduced phase separation propensity compared to wtSFPQ and is up-regulated during neuronal stress

**Fig. 4.**
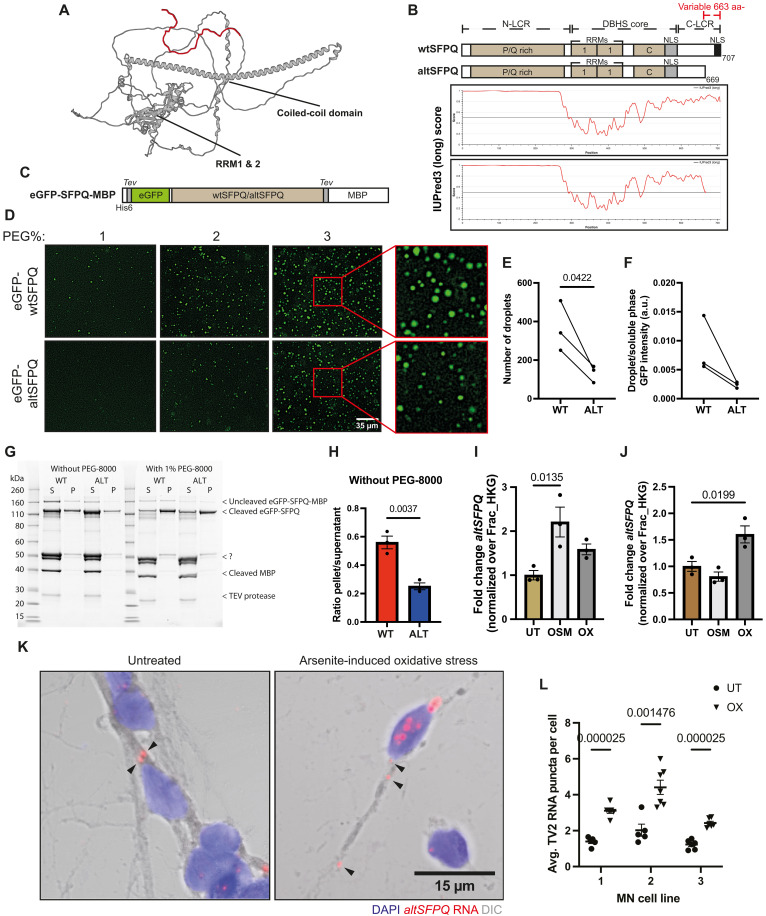
AltSFPQ exhibits reduced phase separation compared to wtSFPQ and is up-regulated during neuronal stress. (**A**) AlphaFold-predicted wtSFPQ protein structure showing the isoform-specific low-complexity C-terminal region (red). (**B**) Schematic depiction of wtSFPQ and altSFPQ protein domains and IUPred disorder prediction graphs. aa, amino acids. (**C**) SFPQ protein purification strategy for LLPS assays. (**D**) Representative images of the eGFP-(wt/alt)SFPQ protein homotypic liquid droplet formation through increasing PEG and fixed protein (3 μM) concentrations, in 150 mM salt buffer; zoomed insets demonstrate spherical forms. (**E**) Quantification of the LLPS droplet number for 3 μM eGFP-(wt/alt)SFPQ proteins in 150 mM salt buffer and 1% PEG (*n* = 3; unpaired *t* test). (**F**) Ratio of GFP intensity inside/outside LLPS droplets for 3 μM eGFP-(wt/alt)SFPQ proteins in 150 mM salt buffer and 1% PEG (*n* = 3; unpaired *t* test). (**G**) Representative image of the sedimentation assay, gel loaded with supernatant and pellet fractions of TEV cleaved eGFP-SFPQ proteins ± PEG. (**H**) Quantification of the pellet/supernatant ratio of cleaved eGFP-SFPQ proteins, for samples processed without PEG (*n* = 3; unpaired *t* test). (**I**) *AltSFPQ* expression (qPCR) in nuclear fractions of motor neurons (DIV6) in response to sorbitol-induced osmotic (OSM) and sodium arsenite–induced oxidative (OX) stresses, normalized over *Nit1* and *NFX1* fraction housekeeping genes (Frac_HKGs). Data are expressed as FC over untreated samples per line and presented as means ± SEM from three control lines; ANOVA with Dunnett’s multiple comparisons. (**J**) As for (I) but in cytoplasmic fractions. (**K**) Representative images of untreated and sodium arsenite–treated motor neurons (DIV6), with BaseScope *altSFPQ* RNA-FISH, DAPI, and bright-field differential interference contrast (DIC) overlay; arrowheads show RNA puncta within axons. (**L**) Quantification of BaseScope RNA-FISH puncta per cell in untreated versus sodium arsenite–induced oxidative stress condition in motor neurons (DIV6); data points reflect fields of view (*n* = 5 to 7), multiple unpaired *t* tests.

As well as subcellular distribution, we reasoned that the altered C-terminal protein sequence may affect the behavior of altSFPQ relative to wtSFPQ protein. AlphaFold prediction suggests that the region unique to wtSFPQ protein (highlighted in red) is unstructured (Fig. 4A). All structured domains common to the DBHS protein family are shared, and the differential C termini are of low complexity. Intriguingly, it was recently reported that the C-terminal low-complexity region (LCR) drives SFPQ liquid-liquid phase separation (LLPS), whereas the N-terminal LCR attenuates this (*46*). The altSFPQ protein loses a large proportion of this C-terminal LCR, reducing its predicted disorder (Fig. 4B). To directly test the relative LLPS propensity of the two proteins, we generated N-terminally enhanced green fluorescent protein (eGFP)–tagged SFPQ proteins with solubilizing C-terminal maltose-binding protein (MBP) tags (Fig. 4C). Recombinant SFPQ proteins were purified from bacterial cultures and concentrated in a storage buffer containing 300 mM NaCl (fig. S5A) at final concentrations of 4.32 mg/ml (GFP-wtSFPQ-MBP) and 4.33 mg/ml (GFP-altSFPQ-MBP); both exhibited high purity (260/280 absorbance ratios of 0.57 for both). For in vitro droplet assays, proteins were diluted to a physiological salt concentration (150 mM NaCl), MBP tags were enzymatically removed, and common crowding agent polyethylene glycol (PEG) was added at increasing concentrations to a fixed 3 μM protein. Micrometer-sized droplets increasingly formed in a PEG concentration–dependent manner (Fig. 4D); both proteins settled to the slide surface as round droplets and fused over time, demonstrative of their liquid-like state (fig. S5B). For quantification, images were taken once droplets had settled to the bottom of the slide but before droplet fusion, enabling quantification of the condensed phase. We quantified droplets and their characteristics from triplicate experiments in the presence of 1% PEG (Fig. 4D and fig. S5C). eGFP-altSFPQ homotypic samples consistently formed fewer droplets with a trend toward reduced size and amount of eGFP protein within droplets compared to eGFP-wtSFPQ (Fig. 4, E and F, and fig. S5). To orthogonally validate our findings, we used a sedimentation assay, whereby samples are centrifuged following tobacco etch virus (TEV) cleavage, and the partitioning of SFPQ proteins into the pelleted fraction is used as a measure of phase separation. A crowding agent increased phase separation of SFPQ proteins, as expected, but protein was also present in the pellet fraction in the absence of any crowding agent. Quantitative comparison of the two SFPQ proteins showed that both without and with a crowding agent altSFPQ exhibited reduced phase separation (Fig. 4, G and H, and fig. S5E). These data demonstrate that, although both SFPQ proteins have LLPS behavior, this is reduced for altSFPQ due to a reduced C-terminal LCR.

LCRs are suggested to be important mediators of functional biocondensation in vivo (*47*). SFPQ is found in cellular stress–induced biocondensates such as cytoplasmic stress granules and nuclear paraspeckles (*48*). We have previously shown that cytoplasmic stress granules form during osmotic and oxidative neuronal stress responses (*49*). Considering divergent LLPS, we hypothesized that altSFPQ expression might be increased during cellular stress. Osmotic (sorbitol) and oxidative (arsenite) stressors were applied to day 6 terminally differentiated hiPSC-derived motor neurons, and *SFPQ* transcript expression was assessed by RT-qPCR. Both stressors caused an ~2-fold increase in *altSFPQ* expression; neither was associated with a significant increase in *wtSFPQ*. Intriguingly, in oxidative and, to a greater extent, osmotic stress, *irSFPQ* levels were significantly reduced (fig. S5, F and G). To gain further insight, we assessed the responses in the nucleus and cytoplasm separately following biochemical fractionation, with the rationale that changes in the nucleus are more likely to arise from posttranscriptional splicing, particularly if coupled to reduced IR, whereas cytoplasmic up-regulation only might indicate stabilization through stress-induced NMD inhibition. In line with the whole-cell analysis, *altSFPQ* expression was significantly increased in the nucleus during osmotic stress, whereas *irSFPQ* was down-regulated (Fig. 4I and fig. S5H). We also quantified the expression of an abundant retained intron derived from the *OGT* gene and its spliced counterpart. This intron is posttranscriptionally spliced out in response to numerous stimuli to enable rapid modulation of O-linked glycosylation (*50*–*52*). The *OGT* protein-coding transcript was increased, whereas the *OGT* intron-retaining transcript is reciprocally down-regulated (fig. S5I), implicating a similar mode of regulation for *irSFPQ* and *altSFPQ* during the hyperosmotic stress response. In the cytoplasm, *altSFPQ* expression was increased in response to oxidative stress only (Fig. 4J); *irSFPQ* was markedly down-regulated in the cytoplasm during osmotic stress (fig. S5J). To orthogonally validate our findings, we performed BaseScope RNA-FISH using 1xZZ probes targeting a unique exon-exon junction for either *wtSFPQ* or *altSFPQ* on fixed day 6 terminally differentiated motor neurons subjected to arsenite-induced oxidative stress. *AltSFPQ* RNAs were detected in cell nuclei, somas, and neurites under untreated and stressed conditions (Fig. 4K). Oxidative stress caused an increase in *altSFPQ* RNA puncta (average of 1.55 RNAs per cell across biological lines to 3.31 under treatment; Fig. 4L) in both the nucleus (average untreated = 0.80, oxidative = 1.82) and extranuclear cell regions (average untreated = 1.00, oxidative = 2.04), broadly in line with qPCR analyses, whereas *wtSFPQ* RNA levels (whole-cell average untreated = 6.20, oxidative = 7.94) were increased in nuclei only (nuclear average untreated = 3.12, oxidative = 5.01; extranuclear untreated = 4.58, oxidative = 5.25) (fig. S6, A and B).

Together, these data demonstrate that the altSFPQ protein exhibits reduced phase separation propensity compared to wtSFPQ, and *altSFPQ* mRNA expression is increased in the context of acute neuronal stresses, raising the possibility that altSFPQ is functional during neuronal stress.

### Altered C terminus drives differential protein binding partners for altSFPQ

**Fig. 5.**
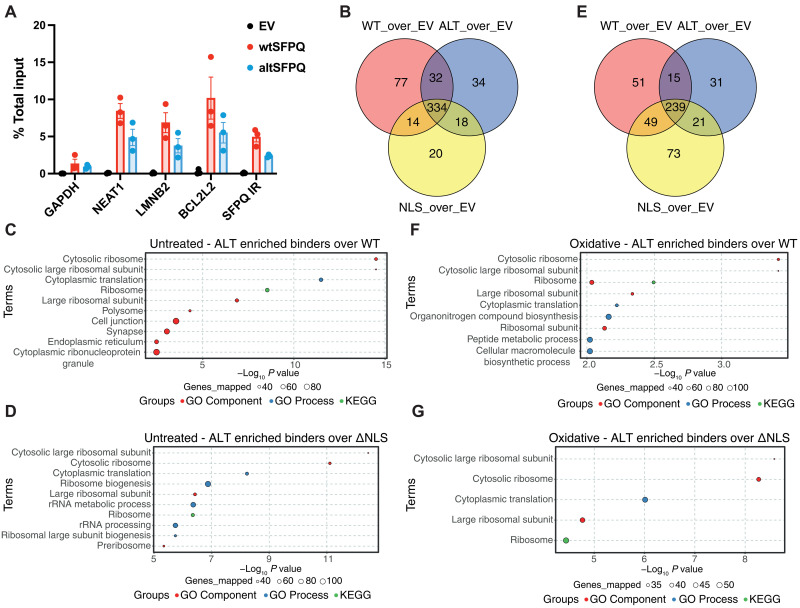
Altered C terminus drives differential protein binding partners for altSFPQ. (**A**) RIP performed on whole-cell lysates from N2A cells expressing HA-SFPQ variants or an EV as a negative control. Levels of associated mRNAs were assessed by qPCR using primers against the indicated targets and expressed as the percentage of total input (*n* = 3; data represent means ± SEM). (**B**) Three-way Venn diagram showing numbers of proteins significantly bound by WT-SFPQ, ALT-SFPQ, and NLS-SFPQ proteins over the EV condition (EV; log_2_FC > 0.5, *P* < 0.05) from affinity purification proteomics data from untreated cells. (**C**) ORA of 808 SFPQ-bound proteins (subsetted based on >0.3 positive correlation with the bait HA-proteins) comparing ALT versus WT binding enrichment from untreated cells (top 10 gene ontology pathways and Kyoto Encyclopedia of Genes and Genomes terms displayed). (**D**) As for (C) but for terms associated with increased ALT-SFPQ over NLS-SFPQ protein binders. (**E**) As for (B) but from oxidative-stressed cells. (**F**) As for (C) in oxidative-stressed cells. (**G**) As for (D) but from oxidative-stressed cells. Some GO terms are shortened.

SFPQ contains two RNA recognition motifs within the core DBHS region of the protein, which mediate its binding to RNAs. It is noteworthy that the altSFPQ protein retains this region entirely, raising the hypothesis that it maintains the same theoretical RNA interactome. To test whether the altSFPQ protein retains capacity to bind the same RNAs in vivo, we performed RNA immunoprecipitation (RIP) following transient expression of the HA-SFPQ variants in neuro-2A (N2A) neuroblastoma cells. altSFPQ binds to established SFPQ RNA interactors: lncRNA (long noncoding RNA) *NEAT1_2* (*53*), *SFPQ retained intron 9* (*25*), and *LMNB2* and *BCL2L2*, which are anterogradely trafficked by SFPQ within axonal granules to synapses (*16*) (Fig. 5A and fig. S7A). A trend toward reduced binding compared to wtSFPQ may be mediated by different levels of these RNAs within the nucleus and cytoplasm, as well as cotranscriptional association of wtSFPQ to these transcripts. Considering the binding of altSFPQ to these RNAs, and that RNA binding can modulate protein biocondensation, we assessed the impact of RNA addition on in vitro phase separation. Addition of an 800–base pair (bp) region of *SFPQ intron 9* induced visible biocondensate formation for both wtSFPQ and altSFPQ proteins in the absence of any crowding agent; this took the form of “beads-on-a-string” fibrillar structures rather than globular droplets (fig. S7B). Furthermore, RNA addition increased sedimentation of both proteins similarly in vitro (fig. S7, C and D). These results suggest that RNA binding enhances the propensity for both wtSFPQ and altSFPQ to form biomolecular condensates, which might alter the nucleocytoplasmic or subcompartmental distribution of both.

Having established that RNA binding is maintained, we hypothesized that the differential low-complexity C terminus of altSFPQ alters its protein interactome. We performed coimmunoprecipitation (co-IP) on lysates from N2A cells expressing WT- and ALT-SFPQ variants, as well as a ΔNLS-wtSFPQ to infer differential binding not conferred by altered nucleocytoplasmic localization alone; empty vector (EV) expression acted as a negative control. Obligate SFPQ binder NONO was efficiently pulled down with each SFPQ variant, whereas GAPDH protein was not detected (fig. S7E); four replicate experiments were subsequently processed for mass spectrometry (MS). Similar numbers of proteins were identified as significantly increased over EV [*P* < 0.05, log_2_FC (fold change) > 0.5] across variants (457 proteins for WT-SFPQ, 418 for ALT-SFPQ, and 386 for ΔNLS-SFPQ), most of which bind to all three proteins (63.1%; Fig. 5B and table S1). Use of human SFPQ (hSFPQ) bait proteins in mouse cells enabled us to use hSFPQ peptide counts to regress out spurious or artifactual binders and minimize the impact of variability in the amount of immunoprecipitated bait protein between variants and replicates. As a result, a total of 808 proteins were identified as binders of any variant based on a positive correlation cutoff of 0.3 target:bait (table S2). We used this subset to carry out overrepresentation analysis (ORA) [STRING; (*54*)] to determine whether ALT-SFPQ binds to different groups of proteins. Compared to WT-SFPQ, ALT-SFPQ exhibits enriched binding to ribosomal and translation-associated, as well as cell junction and synapse-associated proteins (Fig. 5C), whereas terms enriched for WT-SFPQ include spliceosomal, mRNA splicing, and mitochondrial complex (fig. S7F). ΔNLS-SFPQ also exhibited enriched binding to cytosolic ribosome–associated proteins compared to WT-SFPQ, although to a lesser extent, and reduced binding to splicing/spliceosome proteins, as well as ribosome biogenesis terms but not mitochondrial proteins. Furthermore, when we directly compared ΔNLS to ALT-SFPQ, the most enriched terms in ALT were cytosolic ribosome and translation, whereas splicing and mitochondrial proteins were enriched in the ΔNLS interactome (Fig. 5D and fig. S7G). In addition, considering the increased expression of *altSFPQ* in stress, we subjected N2A cells to sodium arsenite–induced oxidative stress and assessed the protein interactomes of SFPQ variants. NONO was efficiently pulled down with each SFPQ variant under stress, whereas GAPDH protein was once more unbound (fig. S7H). Slightly fewer proteins were identified as significantly increased over EV (*P* < 0.05, log_2_FC > 0.5) compared to untreated samples (354 proteins for WT-SFPQ, 306 for ALT-SFPQ, and 382 for ΔNLS-SFPQ), and almost half of all binders were shared by the three proteins (49.9%; Fig. 5E and table S1). ORA revealed that, during stress, ALT-SFPQ still exhibits enriched binding to ribosomal proteins compared to both WT-SFPQ and ΔNLS-SFPQ proteins (Fig. 5, F and G) and decreased binding to splicing-associated terms (fig. S7, I and J).

Together, these data demonstrate differential protein binding of SFPQ variants, which manifests through not only the divergent nucleocytoplasmic localization of endogenous SFPQ proteins but also differences within the cytoplasm driven by alternative C-terminal protein sequences. These may arise directly from alternative C-terminal protein binding, divergent biocondensation properties, or likely a combination of these.

### *AltSFPQ* mRNA is up-regulated in familial and sporadic ALS iPSMNs

**Fig. 6.**
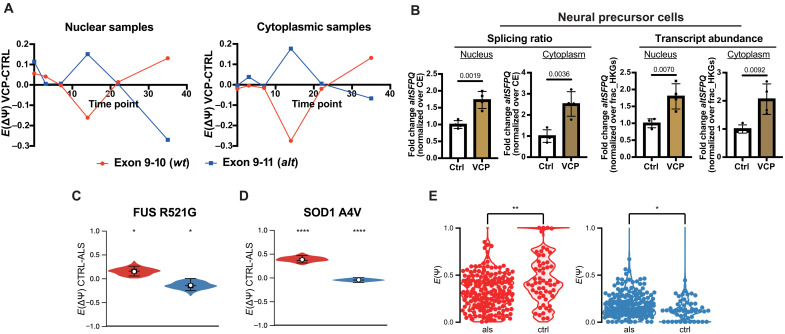
*AltSFPQ* mRNA is up-regulated in familial and sporadic ALS iPSMNs. (**A**) Line graph showing the mean delta PSI value (*VCP* mutant–control) for *wtSFPQ* and *altSFPQ* splicing events across six stages of neuronal differentiation in either nuclear (left) or cytoplasmic (right) fractions derived from four control and four VCP mutant hiPSC lines. (**B**) Bar graphs showing SFPQ exon 9 to 11 splicing levels analyzed by qPCR for NPC nuclear and cytoplasmic fractions from control and *VCP* mutant samples. Splicing was measured by normalizing target expression over the gene expression (constitutive exon) level for each line (left). The right panel depicts the same but for transcript abundance (target normalized over *NIT1* and *NFX1* fraction housekeeping genes). Data expressed as FC over controls and presented as means ± SEM from four lines per group, with data points representing the mean value for each biological line across two technical replicates; unpaired *t* tests. (**C**) Violin plots showing CTRL-FUS delta PSI values (*y* axis) for *SFPQ* splicing events (*wtSFPQ*/exon 9 to 10 in red; *altSFPQ*/exon 9 to 11 in blue) in *FUS* R521G mutant motor neurons versus controls (*55*). (**D**) As for (C) in *SOD1* A4V mutant motor neurons versus controls (*56*). (**E**) Violin plots showing PSI values (*y* axis) for *SFPQ* splicing events (*wtSFPQ*/exon 9 to 10 in red; *altSFPQ*/exon 9 to 11 in blue) in >200 sporadic ALS iPSMNs and >50 control samples (*57*, *58*).

To test whether *SFPQ* is alternatively spliced during motor neuron development in the context of ALS, we examined splicing in our poly(A) RNA-seq dataset of *VCP* mutation–related ALS (*VCP*-ALS hereafter) compared to CTRL samples undergoing motor neurogenesis (*25*), with a focus on LSV chr1:35187001-35187122. Although the broad pattern of splicing in *VCP*-ALS cultures is comparable to that of the controls throughout neurogenesis (fig. S8A), direct comparison between the two groups revealed dysregulation at multiple stages (Fig. 6A and fig. S8, B and C). Aberrant expression of *intron 9*–containing and *wtSFPQ* transcripts has previously been validated (*25*). Increased *altSFPQ* splicing was additionally validated by RT-qPCR, in nuclear and cytoplasmic samples. Furthermore, the abundance of the transcript was found to be increased in both compartments in *VCP*-ALS (Fig. 6B). This represents, to our knowledge, the first report of such altered *SFPQ* splicing in a model derived from patients with ALS.

Having established that *altSFPQ* is increased in *VCP*-related ALS, we next sought to understand whether it is increased across familial ALS. We therefore analyzed publicly available poly(A) RNA-seq datasets of iPSC-derived motor neurons (iPSMNs) from patients with *FUS* or *SOD1* ALS (*55*, *56*). *AltSFPQ* splicing was significantly increased in both familial ALS datasets [*FUS* R521G delta percent spliced in (dPSI) 0.146, *P* = 0.0212; *SOD1* A4V dPSI 0.047, *P* = 4.18 × 10^−5^], whereas *wtSFPQ* splice junction usage was significantly reduced (*FUS* R521G dPSI −0.146, *P* = 0.0212; *SOD1* A4V dPSI −0.378, *P* = 1.66 × 10^−13^) (Fig. 6, C and D); *irSFPQ* was increased in SOD1 A4V neurons (dPSI 0.323, *P* = 5.99 × 10^−12^) (fig. S8D).

To understand the generalizability of *altSFPQ* expression further, we examined sporadic ALS, which comprises 90% of all patients with ALS. To this end, we analyzed *SFPQ* splicing in RNA-seq data from 208 sporadic ALS and 50 control iPSMN lines from combined NeuroLINCS (*57*) (8 sporadic and 8 control lines) and Answer ALS (*58*) (200 sporadic and 42 control lines) studies. It should be noted that these data derive from ribo-depleted but not poly(A) selected RNA libraries. Splicing analysis revealed that the LSV which exhibited the most significant alteration in ALS was the same as in the previous analyses (i.e., exon 9 to 10, exon 9 to 11, and intron 9 inclusive). Splicing of *wtSFPQ* mRNA was markedly reduced in sporadic ALS (dPSI −0.106, *P* = 2.76 × 10^−5^). Conversely, *altSFPQ* splicing (dPSI 0.036, *P* = 2.19 × 10^−3^) was significantly up-regulated (Fig. 6E); IR was also up-regulated (dPSI 0.050, *P* = 5.22 × 10^−4^) (fig. S8E). This result therefore implicates dysregulated *altSFPQ* mRNA expression, along with potential translation, broadly throughout ALS, which may explain the established nuclear loss and cytoplasmic gain of SFPQ protein observed in familial and sporadic postmortem patient motor neurons (*5*).

## DISCUSSION

The nuclear-to-cytoplasmic redistribution of SFPQ is an established phenotype of diseased neurons in ALS. However, the mechanistic basis for this change in subcellular localization has remained undetermined. In this study, we show that, in hiPSC-derived neuronal models of ALS, the *SFPQ* gene undergoes a splicing switch from the canonical protein-coding mRNA (*wtSFPQ*) toward an alternative isoform with a unique 3′ terminal sequence (*altSFPQ*). Despite NMD sensitivity, this isoform undergoes translation to generate a protein omitting a classical NLS sequence (*59*) and exhibiting cytoplasmic-predominant localization, therefore recapitulating the signature redistribution profile found in ALS (*5*). The concept of alternative isoforms leading to a change in subcellular localization of ALS-related RBPs is supported by a recent report of a C terminus truncated short TDP-43 (sTDP-43) isoform that introduces a nuclear export signal and exhibits up-regulation and cytosolic aggregation in a hyperexcitability-dependent manner (*60*). Other C terminus truncated TDP-43 splice isoforms have been identified with similar localization characteristics (*61*). Notably, SFPQ is redistributed in other neurodegenerative diseases such as Alzheimer’s disease and FTD (*18*, *20*, *21*). Whether SFPQ splicing is similarly dysregulated in these other diseases is of notable interest. Our findings are also supported by a recent preprint from Zeng *et al.* (*62*), demonstrating that knockdown of the ALS-associated TDP-43 protein shifts poly(A) site usage from proximal to distal in the *SFPQ* gene, consistent with the shift from *wtSFPQ* to *altSFPQ* expression, which we have identified across models derived from patients with familial and sporadic ALS. This may provide a mechanism contributing to *SFPQ* dysregulation, although SOD1 and FUS fALS should progress by a different mechanism given that these cases typically lack TDP-43 pathology.

Seminal work identified SFPQ (PSF as it was previously known) as a spliceosome-associated nuclear protein and described multiple SFPQ transcript isoforms including one consistent with *altSFPQ* mRNA (*26*, *43*, *63*). However, although proteolytically truncated SFPQ proteins have been identified in specific contexts (*64*), no alternative splicing determined proteoform has been demonstrated to the best of our knowledge. Our data suggest that, under normal conditions, *wtSFPQ* mRNA expression markedly outweighs that of *altSFPQ*. Consistent with this, SFPQ is typically observed as a highly expressed nuclear protein wherein it carries out numerous functions including transcriptional regulation, splicing and polyadenylation, DNA damage repair, and paraspeckle formation (*6*, *65*). Nonetheless, cytoplasmic functionality of SFPQ was recently reported in neurons, including roles in mRNA transport (*15*, *16*, *66*). Furthermore, axonal developmental defects in zebrafish conferred by total loss of SFPQ are at least partially rescued by a ΔNLS cytoplasmic SFPQ protein (*17*). Although altSFPQ loses a classical NLS, it retains two RNA recognition motifs, a NonA/paraspeckles (NOPS) domain, and a coiled-coil domain (*67*). We have demonstrated that it retains capacity to bind established SFPQ RNA targets, including those with which it associates in axonal RNA transport granules (*16*). Gene distal alternative last exon isoforms broadly display preferential localization to neurites (*68*), promoting isoform-specific axonal translation and formation of a distinct translatome (*69*). We have observed *altSFPQ* mRNA localization within neurites of hiPSC-derived motor neurons; it follows that *altSFPQ* may undergo local translation to carry out known SFPQ functions in axonal and synaptic maintenance (*17*). Notably, low-level expression of an ALS-linked C-terminally truncated FUS protein, which thereby exhibits cytoplasmic localization, causes marked changes in the spinal cord transcriptome (*70*), implicating small changes in cytoplasmic SFPQ expression as potentially impactful. However, the contribution of increased *altSFPQ* to ALS pathogenesis via a cytoplasmic gain of function is currently undetermined.

High conservation of the *altSFPQ* first alternative exon implies important functionality. The two additional downstream exons display much less conservation. Considering that its NMD sensitivity is likely conferred by the presence of downstream introns within the 3′ untranslated region, it is possible that, in lower-order organisms, the alternative protein is expressed from a relatively stable transcript, assuming that lower conservation predicts lower usage. As such, the protein function of the altSFPQ isoform may have preceded a more complex role involving autoregulation as well as alternative protein localization and function. Of note, although PTC presence is generally predictive of NMD sensitivity, detectable protein can still be generated from such transcripts despite their low levels (*71*).

In addition to a structured central region, SFPQ has low-complexity N- and C-terminal regions, the latter of which drives its LLPS (*46*). AltSFPQ lacks a large proportion of the C-terminal LCR present in wtSFPQ. We predicted that this might confer reduced phase separation propensity and confirmed as such in vitro. Alternative isoforms exhibiting differential phase separation mediated by differential LCR inclusion have been demonstrated for at least one other gene (*72*), supporting our model whereby alternative splicing regulates SFPQ protein biocondensation properties and thereby presumably its cytoplasmic function. LLPS droplets of ALS-relevant RBPs may transition into aggregates with time (*73*, *74*). The biocondensates (spherical droplets) we observed in our phase separation studies for both SFPQ proteins did not completely fuse once settled to the slide surfaces, thereby resulting in the appearance of many “deformed” spherical structures. This is consistent with initial LLPS formation, which progresses to a viscous liquid-like or even a gel-like state (*75*); this has been directly related to progression toward pathological fibrillization in the context of other RBPs relevant to ALS (*76*). The degree to which this state is reversible has not been examined in this study, but it is noteworthy that cytoplasmic aggregates containing SFPQ are sometimes observed in ALS postmortem tissue (*24*). AltSFPQ does not lack biocondensate properties but rather displays reduced propensity to form biocondensates compared to wtSFPQ. As such, whereas differential biocondensation proclivity may subserve differential functions, increased altSFPQ in ALS over time may still lead to the formation of cytoplasmic aggregates or contribute to an “aggregation-prone” environment for other proteins. On the other hand, the low-level diffuse cytoplasmic signal, rather than aggregates, also described for SFPQ in ALS pathology is also consistent with altSFPQ protein expression.

Mechanisms underlying direct wtSFPQ nuclear-to-cytoplasmic relocalization have been described (*22*); these may occur alongside altSFPQ expression or represent context-specific requirements for the cytoplasmic localization of SFPQ proteins with different behaviors and thereby functions. In line with this, we observed increased *altSFPQ* expression, at least partially independent of *wtSFPQ* changes during acute neuronal stress responses. Furthermore, we identified divergent classes of protein binding partners for the different SFPQ proteins. Some of these are likely conferred by the differential nuclear-to-cytoplasmic localization of wtSFPQ versus altSFPQ in the contexts that we have assessed. However, differential binding of altSFPQ and ΔNLS-SFPQ to ribosomal large subunit proteins, as an example, implies divergent behavior within the cytoplasm. This may be a direct consequence of the differential C-terminal sequences conferring different binding, reduced stochastic low-complexity binding, altered biocondensation, or a combination. In line with this, despite potential assay-related biocondensate disruption, it was intriguing to observe reduced binding of altSFPQ to mitochondrial proteins, which were previously found to interact with the RBP FUS in an LLPS-dependent manner (*74*). In addition, SFPQ has previously been identified as a binder of the large ribosomal subunit in a large omics study (*77*); it is plausible that this might have been altSFPQ, considering that altSFPQ peptides would be indistinguishable from those of wtSFPQ. Whether increased binding of altSFPQ to the ribosome confers altered translation in vivo is an interesting outstanding question, particularly considering translation defects identified in ALS (*78*).

AltSFPQ readily interacted with endogenous SFPQ in our dataset, implying in vivo heterodimerization; SFPQ and the other DBHS proteins form both homo- and heterodimers with one another (*44*). Furthermore, coexpression of wtSFPQ and altSFPQ proteins resulted in subcellular redistribution of both, with clear signal colocalization in the cytoplasm, whereas the overexpressed altSFPQ protein caused redistribution of endogenous NONO toward the cytoplasm. DBHS domain–containing proteins in the dipteran *Chironomus tentans* regulate one another’s subcellular localization (*79*), which raises the intriguing prospect of human DBHS protein heterodimerization regulating subcellular localization of SFPQ proteins, as well as NONO and possibly PSPC1. The relative impact will depend on expression level and heterodimer affinities, which are nonuniform across different cells and tissues (*65*). In the context of ALS, this implies that expression of altSFPQ will not immediately “drag” wtSFPQ from the nucleus, but as dysregulated isoform expression shifts increasingly toward altSFPQ during the course of disease, this will progressively affect nuclear wtSFPQ levels through direct protein-protein interactions. As well as subcellular localization, it follows that altSFPQ dimerization with DBHS proteins might regulate their biocondensation, given that intrinsically disordered protein composition determines phase separation and aggregation propensity of these proteins (*46*), and thereby alter their molecular functions (*80*). We have also described the formation of fibrillar structures, rather than droplets, upon addition of RNA to eGFP-SFPQ proteins in vitro. A similar behavior has been observed for NONO and PSPC1 proteins (*80*, *81*) but not FUS: RNA initially enhances the formation of globular droplets, until high levels of RNA are added, whereupon droplets are solubilized (*82*). This implies a shared mechanism by which RNA-dependent modulation of biocondensation regulates DBHS protein function, including altSFPQ.

Our stress data raise the possibility that *altSFPQ* derives from a reservoir of “poised” *SFPQ* intron-retaining transcripts, which we and others have previously identified as increased in expression in familial and sporadic ALS (*5*, *24*, *25*). Broadly, stress-responsive splicing out of normally retained introns has been proposed as a mechanism for cellular adaptation to stress (*50*, *83*, *84*). Candidate-based single-molecule fluorescence in situ hybridization (smFISH) studies have suggested that alternatively spliced exons are posttranscriptionally spliced at a greater frequency than their constitutively spliced counterparts (*85*). This possibility has been orthogonally supported by metabolic labeling studies using 4-thiouridine to identify and isolate RNA within minutes of transcription, which revealed that alternative splicing is slower than constitutive splicing (*86*). Further evidence supporting this phenomenon whereby a reservoir of IR transcripts are “poised” and then posttranscriptionally spliced in a stimulus-specific manner comes from live-cell reporters, which have convincingly demonstrated that introns with relatively weak splice sites are removed over a longer time frame compared to introns with stronger and canonical splice sites (*87*). Additionally, retained introns are more evolutionarily conserved than their constitutive counterparts, which is consistent with functional significance (*50*).

**Fig. 7.**
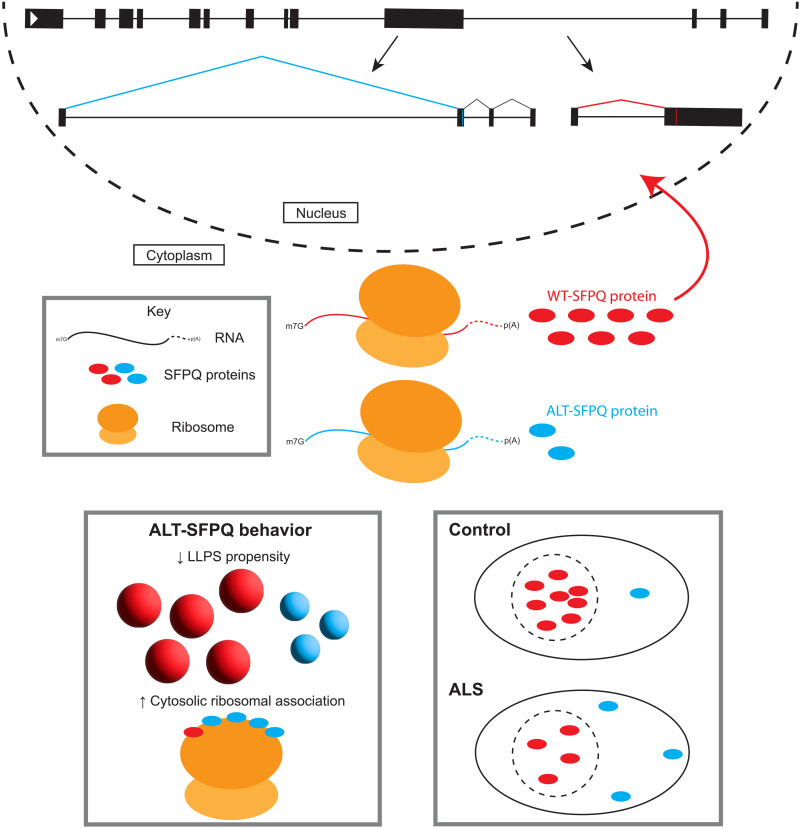
Proposed model of SFPQ protein redistribution in ALS. Under control conditions, the *SFPQ* gene expresses at least two splicing isoforms; *wtSFPQ* (red RNA) encodes a nuclear predominant protein, the other (*altSFPQ*; blue RNA) is largely degraded but encodes a primarily cytoplasmic protein. The altSFPQ protein exhibits reduced phase separation propensity and partially distinct protein interactome, including translation-associated machinery. In ALS models, *wtSFPQ* is reduced whereas *altSFPQ* is increased, possibly resulting in reduced nuclear and increased cytoplasmic SFPQ proteins, manifesting as the nuclear-to-cytoplasmic redistribution of total SFPQ protein in disease.

Together, we have highlighted dysregulated splicing of *SFPQ* in ALS, the translation potential of a previously identified mRNA isoform, and present this as a potential mechanism driving the nuclear loss and cytoplasmic gain of SFPQ protein in this disease (Fig. 7). Many questions remain, including the extent of altSFPQ pathology in ALS, the generalizability of altered SFPQ splicing in neurodegeneration, the functions of altSFPQ, and whether these are cell type- and context-specific. Further investigations addressing these points will determine the potential therapeutic benefit of targeting this SFPQ splicing isoform in ALS.

## MATERIALS AND METHODS

### Experimental models

#### Ethics statement

Informed consent was obtained from healthy control donors and patients in this study. Experimental protocols were enacted according to regulations and guidelines outlined by UCLH’s National Hospital for Neurology and Neurosurgery and UCL’s Institute of Neurology Joint Research Ethics Committee (09/0272).

#### hiPSCs and motor neurons

hiPSC lines used are detailed in table S3. Three control lines are commercially available: Coriell (ND41866*C), Thermo Fisher Scientific (A18945), and Cedars Sinai (CS02iCTR-NTn4). HiPSCs were maintained on Geltrex (Life Technologies) with Essential 8 media (Life Technologies) and passaged using EDTA (Life Technologies, 0.5 mM). Directed differentiation from hiPSCs to motor neurons was performed using a previously published protocol (*88*). Briefly, hiPSCs were first grown to 100% confluency and then differentiated to the neuroepithelium in chemically defined medium consisting of Dulbecco’s modified Eagle’s medium (DMEM)/F12 GlutaMAX, Neurobasal, l-glutamine, N2 supplement, nonessential amino acids, B27 supplement, β-mercaptoethanol (βME) (all from Life Technologies) and insulin (Sigma-Aldrich). Treatment with small molecules from days 0 to 7 was as follows: 1 μM dorsomorphin (Millipore), 2 μM SB431542 (Tocris Bioscience), and 3.3 μM CHIR99021 (Miltenyi Biotec). Starting from day 8, the neuroepithelial layer was patterned for 7 days with 0.5 μM retinoic acid and 1 μM purmorphamine. At day 14, NPCs were treated with 0.1 μM purmorphamine for a further 4 days before being terminally differentiated in 0.1 μM compound E (Enzo Life Sciences) to promote cell cycle exit. All cultures were maintained at 37°C and 5% CO_2_.

For RNA interference, NPCs were plated onto 12-well plates in N2B27 media at density of 5 × 10^5^ cells per well and then transfected with 30 pmol of siRNA targeting SFPQ (pool of four siRNAs, ON-TARGETplus SmartPool SFPQ siRNA, L-006455-00-0005, Horizon) or nontargeting control siRNA (pool of four siRNAs, ON-TARGETplus Non-targeting Control Pool, D-001810-10-05, Horizon). Two microliters of Lipofectamine RNAiMax was used as a transfection reagent according to the manufacturer’s protocol. Following overnight incubation, cell media were changed to fresh N2B27 neural media supplemented with 0.1 μM compound E to push cells into terminal differentiation. After 3 days in this medium (96 hours posttransfection), samples were harvested. For CHX treatments, CHX was added directly to growth media at a final concentration of 100 μg/ml and cells were maintained in culture for 6 hours. For SMG1 inhibition, 0.5 μM hSMG-1 inhibitor 11e (a kind gift from O. Muehlemann, University of Bern) was added directly to growth media on cells and cells were maintained in culture for 24 hours. For CHX and SMG1i treatments, dimethyl sulfoxide (DMSO) was used as the vehicle control. For osmotic stress experiments, 0.4 M sorbitol was added onto cells in normal motor neuronal media and incubated for 1 hour in a standard 37°C incubator. For oxidative stress experiments, 0.5 mM sodium arsenite (Sigma-Aldrich) was added, again for 1 hour at 37°C. Treatment compounds were diluted in cell media. Cells from each condition plus untreated cultures were then harvested.

#### HEK293T, HeLa, and N2A cell lines

The HEK293T, HeLa, and N2A lines were obtained from the Francis Crick Cell Services facility, following mycoplasma testing. Cells were cultured in DMEM containing 10% fetal bovine serum and penicillin/streptomycin and grown at 37°C and 5% CO_2_. Cells were transfected with Lipofectamine 2000 in Opti-MEM according to the manufacturers’ guidelines. For CHX treatments, CHX was added directly to growth media at a final concentration of 100 μg/ml and cells were maintained in culture for 6 hours. For SMG1 inhibition, 1 μM SMG1i was added directly to growth media on cells and cells were maintained in culture for 24 hours. DMSO was used as the vehicle control for CHX and SMG1i treatments. For DRB (Sigma-Aldrich) treatments, DRB was added directly to growth media at a final concentration of 75 nM and cells were maintained in culture for indicated times. For alpha-amanitin treatments, alpha-amanitin (Roche) was added directly to growth media at a final concentration of 5 μg/ml and cells were maintained in culture for indicated times.

#### iNeuron cell culture

Stably expressing doxycycline-inducible BS6 2H9 *Neurogenin2* (*Ngn2*) i3-iPS cells were used for rapid differentiation into cortical neurons (i3Neurons) using a previously described method (*89*). Briefly, i3iPS cells were grown to 80% confluency, washed with phosphate-buffered saline (PBS), lifted with Accutase (Gibco), and plated at 1 × 10^7^ cells in a 10-cm Geltrex-coated dish (DIV0). Cells were maintained from DIV0–3 in an induction medium consisting of DMEM/F12 (Gibco), 1× N2 (Thermo Fisher Scientific), 1× GlutaMAX (Gibco), 1× Hepes (Gibco), 1× nonessential amino acids (Gibco), doxycycline (2 μg ml^−1^), and 10 μM Y-27632 (DIV0 only; Tocris), which was exchanged daily. On DIV3, cells were dissociated with Accutase and replated on to poly(ethylenimine)-coated (Sigma-Aldrich) and laminin-coated (Sigma-Aldrich) 12-well plates at 7.5 × 10^5^ cells per well in neuronal maintenance medium consisting of Neurobasal medium (Gibco), supplemented with 1× B27 (Gibco), brain-derived neurotrophic factor (10 ng ml^−1^; PeproTech), NT-3 (10 ng ml^−1^; PeproTech), laminin (1 μg ml^−1^), and 5-fluor-2′-deoxyuridine (FDU; 2 μM DIV4, 0.5 μM thereafter; Gibco). From DIV3 to DIV14, cells were maintained in neuronal maintenance medium. Lentiviral transduction (multiplicity of infection: 7) to overexpress SFPQ-T2A-mAPPLE proteins was carried out 1 hour after DIV3 replating, with a full media change carried out the next day. On day 11 posttransduction (DIV14 overall), cells were washed once in PBS, then lifted with EDTA, pelleted at 2000*g* for 5 min at 4°C, and then processed for RNA extraction.

### Method details

#### Generation of plasmids

pcDNA3.1-HA-SFPQ was a gift from R. Segal (Addgene plasmid no. 166959; http://n2t.net/addgene:166959; RRID:Addgene_166959). For the HA-altSFPQ plasmid, a unique C-terminal altSFPQ coding sequence was amplified from HEK293T cDNA with esp3I and ecoRI overhangs and cloned into the HA-SFPQ plasmid with the esp3I-ecoRI insert removed. For the HA-NLS-SFPQ plasmid, a portion of the wtSFPQ coding sequence up to, but not including, the terminal nuclear localization sequence was amplified from HEK293T cDNA with esp3I and ecoRI overhangs and cloned into the HA-SFPQ plasmid with the esp3I-ecoRI insert removed. For the EV plasmid, a multiple cloning site with kpn1-ecoRI overhangs was cloned into the HA-SFPQ plasmid with the kpn1-ecoRI insert (entire HA-wtSFPQ sequence) removed. eGFP-wtSFPQ and mAPPLE-altSFPQ expression constructs were generated by cloning eGFP or mAPPLE sequences into pcDNA3.1-HA-(wt/alt)SFPQ plasmids using NEBuilder (NEB).

To generate His6-*Tev*-eGFP-SFPQ-*Tev*-MBP plasmids ORF (open reading frame) codon optimized for *Escherichia coli*, wtSFPQ and altSFPQ coding sequences (GeneArt, Thermo Fisher Scientific) were cloned into pMal-C5-*Tev*-eGFP-*Tev*--His6 (generated by gene synthesis and cloning of *Tev*-eGFP-*Tev*-His6 cDNA into SacI-HindIII sites of pMalC5x, General Biosystems) using XbaI and BamHI restriction sites to generate pMal-C5-*Tev*-SFPQ-eGFP-*Tev-*-His6. pMal-C5-*Tev*-SFPQ-eGFP-*Tev*--His6 was then used to PCR and then combine the following four fragments using Gibson assembly (NEB): ATG-His6-*TEV*-GFP-SFPQ, (wt/alt)SFPQ-*TEV*-MBP, MBP-STOP, and MBP-STOP-vector_backbone-ATG-His6-*TEV*-GFP, giving final His6-*Tev*-eGFP-SFPQ-*Tev*--MBP constructs. For in vitro transcription of *SFPQ intron 9* RNA, an 800-bp region (GTAATGTATCC-UGAAUGUGGAU), which is bound by SFPQ (*5*), was generated by gene synthesis and cloned into a pMA vector, with a T3 promoter upstream and an EcoRV restriction site downstream of the *SFPQ* intron sequence (GeneArt, Thermo Fisher Scientific).

#### Lentivirus production

A pCDH-hSYN lentiviral plasmid was used as the vector to create the control and SFPQ lentiviral constructs. Subcloning of SFPQ constructs was undertaken as a three-fragment insertion of the (i) HA-SFPQ coding sequence, (ii) T2A, and (iii) mAPPLE using NEBuilder (NEB) to yield final pCDH-hSYN-HA_SFPQ-T2A-mAPPLE products. Control was generated by cloning the mAPPLE only into the vector to generate the pCDH-hSYN-mAPPLE plasmid.

Third-generation VSV-G lentiviruses were prepared in HEK293T cells: SFPQ or control lentiviral transfer plasmids transfected with pLP1, pLP2, and pVSV-G lentiviral packaging and envelope plasmids, using Lipofectamine 2000 (Thermo Fisher Scientific). Virus-containing supernatants were collected 48 and 72 hours posttransfection, combined, and concentrated by precipitation with PEG-8000–NaCl (final 8% and 80 mM, respectively) for >4 hours at 4°C. Virus containing pellets were resuspended in PBS and titered using ddPCR (from Addgene; https://addgene.org/protocols/lentivirus-ddpcr-titration/) and then aliquoted and stored at −80°C until use.

#### Recombinant protein expression and purification

Expression and purification of recombinant His6-eGFP-SFPQ-MBP (wt and alt) were performed using a protocol adapted from (*90*). In brief, the bacterial expression vectors were transformed into Rosetta-2(DE3)-pLysS *E. coli* and grown in standard Lysogeny broth (LB) medium supplemented with chloramphenicol (33 μg/ml) and carbenicillin (100 μg/ml). At an optical density at 600 nm (OD_600_) of ∼0.8, cells were induced with 0.1 mM IPTG (isopropyl-β-D-thiogalactopyranoside) for 24 hours at 12°C. Cells were lysed by sonication (5 x 1-min pulses with 5-min breaks between pulses) in lysis buffer [50 mM Na_2_HPO_4_/NaH_2_PO_4_ (pH 8.0), 300 mM NaCl, 20 μM ZnCl_2_, 20 mM imidazole, and 10% glycerol] supplemented with 4 mM βME, DNase1 (20 U/ml; Roche), and RNase A (200 μg/ml; Sigma-Aldrich), and the debris was removed by centrifugation at 24,500*g* and 10°C for 1 hour. The cleared lysates were then incubated with Ni-NTA agarose beads (Qiagen) and washed with lysis buffer before elution in HisTrap buffer [50 mM Na_2_HPO_4_/NaH_2_PO_4_ (pH 8.0), 300 mM NaCl, 20 μM ZnCl_2_, 250 mM imidazole, and 4 mM βME]. The eluate was then incubated with amylose resin (NEB), washed with wash buffer [50 mM Na_2_HPO_4_/NaH_2_PO_4_ (pH 8.0), 300 mM NaCl, 20 μM ZnCl_2_, 20 mM imidazole, and 4 mM βME], and subsequently eluted in wash buffer (+ 20 mM maltose). Last, proteins were concentrated using 100-kDa molecular weight cut-off Viva spin concentrator columns, then aliquoted, and stored at −80°C for one time use per aliquot. Protein concentrations were determined from their absorbance at 280 nm using ε predicted by the ProtParam tool; 260-/280-nm ratios of all purified proteins were ∼0.6.

#### In vitro phase separation assays

Purified full-length His6-eGFP-SFPQ-MBP stock proteins (wt or alt) were centrifuged at 17,000*g* for 10 min at 4°C to remove the precipitated/aggregated protein, and then protein concentrations were reestablished by measuring the absorbance at 280 nm by NanoDrop using the respective extinction coefficients (ε). Samples were then diluted in salt-free droplet buffer [50 mM Na_2_HPO_4_/NaH_2_PO_4_ (pH 8.0) and 20 μM ZnCl_2_] to a final 150 mM NaCl concentration.

For droplet assays, phase separation was induced by addition of TEV protease (Sigma-Aldrich) at 25°C for 60 min, followed by addition of PEG-8000 crowding agent and transfer to μ-slide 18-well–flat chambers (IBIDI). Imaging for quantification of eGFP-tagged SFPQ proteins was performed by wide-field fluorescence microscopy; one 20x magnification image was taken per condition in each experimental batch. Flat-field illumination correction, filtering, noise reduction, object identification, and output measurements were performed with CellProfiler. Imaging for figs. S4B and S6B were taken on an 880 laser scanning confocal microscope (Zeiss) at 63x objective.

For in vitro sedimentation assays, samples were processed as described in (*91*). Briefly, the TEV protease was added and the sample was incubated at 30°C for 2 hours to cleave the MBP tag and then incubated a further 30 min at room temperature with or without addition of PEG-8000 crowding agent. For experiments measuring the impact of RNA addition, an 800-bp section of *SFPQ intron 9* was added for 30 min at room temperature without a crowding agent, following MBP cleavage. The RNA was in vitro transcribed from T3-*SFPQintron9* using the T3 MEGAscript kit (Thermo Fisher Scientific) following linearization of pMA-T3-*SFPQintron9*-EcoRV by EcoRV-HF restriction enzyme digest and phenol/chloroform cleanup; the reaction was incubated with TurboDNAse for 30 min at 37°C after transcription, RNA was precipitated by phenol/chloroform, and a single 800-bp product was confirmed by running on an agarose gel before use. After 30 min, room temperature incubation samples were centrifuged for 15 min at 17,000*g* at 4°C to pellet-formed condensates. Supernatant fractions were collected, and pellets were resuspended in the same volume of 150 mM NaCl droplet buffer; a 4x NuPAGE LDS sample buffer and 50 mM dithiothreitol (DTT) final concentration were added, and protein samples were denatured at 80°C for 10 min. Samples were equally loaded onto Invitrogen NuPAGE 4 to 12% Bis-Tris gels, along with an unstained protein ladder, and standard SDS-PAGE runs proceeded. Gels were then fixed for 30 min in 50% ethanol and 10% acetic acid and then stained overnight (SYPRO Ruby). The following day, gels were destained with 10% ethanol and 10% acetic acid and then imaged on a GelDoc XR+ (Bio-Rad). Intensities of cleaved, full-length eGFP protein bands were quantified in ImageJ.

#### Cell fractionation

Biochemical subcellular fractionation was achieved using the Ambion PARIS kit cell fractionation buffer, following the manufacturer’s general protocol, and an 8 M urea nuclear lysis buffer was prepared in-house. Initially, cells were washed once using ice-cold PBS. Cytosolic fraction was then obtained by lysing cells directly in ice-cold cell fractionation buffer for 10 min, thus disrupting plasma membranes while leaving nuclear membranes intact. Lysates were then centrifuged for 3 min at 500*g* and 4°C. The supernatant was then collected and further centrifuged at maximum speed and 4°C, and the resulting supernatant was then processed as cytosolic fraction. Nuclear pellets from the first centrifugation step were washed once with cell fractionation buffer and then lysed on ice for 30 min in 8 M urea nuclear lysis buffer, containing 50 mM tris-HCl (pH 8), 100 mM NaCl, 0.1% SDS, and 1 mM DTT. The resulting nuclear fraction was then homogenized using a QIAshredder (QIAGEN) to shred chromatin and reduce viscosity, before being further processed for RNA extraction. Both lysis buffers were supplemented with RiboLock RNase Inhibitor (0.1 U/μl) and Halt Protease Inhibitor Cocktail.

#### Northern blotting

For *altSFPQ* Northern blot analysis, 5 μg of total RNA per condition was denatured and electrophoresed on a 1% formaldehyde-agarose gel for 2 hours at 80 V. Following washes in RNase-free H_2_O and 20x SSC buffer, the blot was transferred onto a Hybond nylon membrane (Amersham Biosciences) overnight via capillary transfer at room temperature. The next day, RNA was cross-linked onto the membrane by ultraviolet light (254 nm) exposure at 120 J. Then, the membrane was blocked in prehybridization buffer (Sigma-Aldrich) plus preboiled salmon sperm single-stranded DNA (ssDNA) (100 μg/ml) at 68°C for 4 hours, following which the prehybridization solution was replaced by PerfectHyb Plus Hybridization Buffer (Sigma-Aldrich) [plus denatured ssDNA (100 μg/ml)]. A purified probe was added, and the membrane was incubated at 68°C overnight. Probes were synthesized as follows: The target region was amplified from HEK293T cDNA using 5′-3′ (TGATTGATGTTGGCTGATATTGGA) and 3′-5′ (ACGTTCATTCCTCCTTCCACT) primers and standard PCR. Probes were labeled using the Random Primer DNA Labeling Kit v2 (Takara), according to the manufacturer’s instructions. Briefly, 20 ng of the probe (template), 2 μl of a random primer, and nuclease-free water were combined in a 14-μl reaction, which was then heated at 95°C for 3 min to denature template/primers. To this reaction, the following was added: 2.5 μl of 10x buffer, 2.5 μl of a deoxynucleoside triphosphate mixture [containing 0.2 mM dGTP (deoxyguanosine triphosphate), dATP (deoxyadenosine triphosphate), and dTTP (deoxythymidine triphosphate) each] and 5 μl of labeled deoxycytidine triphosphate (dCTP) (32P-dCTP, 9.25 megabecquerels). Last, following the addition of 1 μl of exo-free Klenow fragment (2 U/μl), the reaction was incubated at 37°C for 2 hours. Labeled probes were then purified using MiniQuick Spin DNA Columns (Roche) according to the manufacturer’s instructions. The purified probe was denatured by heating at 100°C for 5 min before addition to the hybridization buffer.

On the following day, the prehybridization solution was removed and the membrane was washed three times in 2x SSC and 0.1% SDS at 68°C, followed by final high stringency wash in 0.2x SSC and 0.1% SDS. The hybridized membrane was then exposed to a storage phosphor screen for a few hours, capturing the hybridized signal, and then developed using a Molecular Imager FX Pro Plus System and Quantity One 1-D software (Bio-Rad).

#### Polysome profiling

Sucrose gradients (10 to 50%) were prepared by mixing 10 and 50% sucrose solutions [prepared in 20 mM tris-acetate (pH 7.5), 150 mM NaCl, and 4 mM MgCl_2_], using a Biocomp Gradient Master Base Unit (Wolf Laboratories), according to the manufacturer’s instructions. Before usage, gradients were stored at 4°C. Cells were cultured until ~70 to 80% confluent, unless alternative confluency was unavoidable (i.e., neural induction), and then washed once in ice-cold PBS + CHX (100 μg/ml) before lysis in lysis buffer [20 mM tris-HCl, 150 mM NaCl, 4 mM MgCl_2_, 1% Triton X-100, 1 mM DTT, 1x protease inhibitor cocktail (EDTA-free), 1x RNase inhibitor (RiboLock and SUPERase·In), and CHX (100 μg/ml)]. Nuclei were cleared with a brief centrifugation at 500*g*; the supernatant was then further cleared by centrifugation at max speed to remove any insoluble material. In the case of EDTA treatment, 30 mM EDTA was added to the cleared lysate and incubated on ice for 30 min. Samples, containing equal quantities of RNA, quantified using the QuantiFluor RNA system (Promega), were then loaded onto the sucrose gradient and subjected to ultracentrifugation in a Beckman SW41 rotor at 39,000 rpm for 1.5 hours at 4°C. Fractions were collected using the Biocomp machine (Wolf Laboratories) and on-screen profiles to guide manual dispensal. Overnight RNA precipitation using 0.1 volume of 3 M sodium acetate and 2.5 volumes of 100% ethanol was followed by Turbo DNase (Invitrogen) treatment and lastly TRIzol:chloroform RNA extraction.

#### Splice-modulating ASOs

Before experimentation, all ASOs were dissolved in nuclease-free H_2_O and the concentration was determined by OD_260_ absorbance. PS backbone–, 2′MOE-modified 18-nucleotide ASOs were designed (IONIS Pharmaceuticals) to target the SFPQ gene and modulate splicing through steric blocking, without recruitment of RNase H.

For the ASO screen, HEK293T cells were seeded onto 24-well plates such that the confluency was around 30% the next day. Lipofectamine 2000 and Opti-MEM were used, according to the manufacturer’s guidelines, to transfect 200 nM ASOs, and cells were harvested 48 hours after transfection. Expression levels for target mRNAs were compared to mock treatment in which Lipofectamine reagent, but no ASO, was introduced. For follow-up experiments using a candidate ASO, nontargeting control (NC2; IDT) was additionally included, having the same nucleotide length and chemistry, to account for nonspecific effects.

#### RNA extraction and RT-qPCR

Cells were washed with PBS, and then total RNA was typically extracted using the Maxwell RSC simplyRNA Cells kit (Promega), using associated reagents including DNase treatment and a Maxwell RSC 48 machine, unless otherwise specified in associated methods. RNA concentrations were determined via NanoDrop; 260/280 and 260/230 ratios provided estimates of purity. Total RNA was reverse transcribed using the RevertAid First Strand cDNA synthesis kit (Thermo Fisher Scientific) or Superscript IV (Thermo Fisher Scientific) using random hexamer primers. qPCR was performed using the PowerUP SYBR Green Master Mix (Thermo Fisher Scientific) and the QuantStudio Flex Real-Time PCR System (Applied Biosystems). Primers used are listed in table S4. Before experimental use all primers used underwent quality control testing, involving assessment of melting curves, amplification characteristics, and agarose gel electrophoresis of the PCR product. Within each experiment, an RT-minus sample was used as a negative control. The relative transcript expression level was determined through normalization of target expression to that of a housekeeping gene(s); where multiple housekeeping genes were used, the geometric mean of these is used for normalization. “Proportional transcript isoform expression” (e.g., percent spliced in/splicing ratio) was determined through normalization of the target isoform to the expression of a constitutively expressed region within the host gene. Data were analyzed according to the ddCt method, typically expressed as FC over the control/untreated group.

#### Protein extraction, quantification, and Western blotting

To extract total protein for Western blotting, cells were lysed in RIPA (radioimmunoprecipitation assay) buffer [50 mM tris-HCl (pH 7.4), 150 mM NaCl, 0.5% sodium deoxycholate, and 1% NP-40], supplemented with Halt Protease Inhibitor Cocktail for 15 min on ice, followed by 10 cycles of sonication (Bioruptor) with alternating 30-s on/off. Following a further 15-min incubation on ice, lysates were cleared by centrifugation at max speed and 4°C. The protein was quantified using DC Assay (Bio-Rad) as per the manufacturer’s instructions before downstream processing. When necessary, protein samples were concentrated using methanol-chloroform precipitation. A 4x NuPAGE LDS sample buffer and 50 mM DTT final concentration was added to the protein sample and placed at 70°C for 10 min for denaturation. Samples were loaded onto Invitrogen NuPAGE 4 to 12% Bis-Tris or 7% tris-acetate protein gels and run at 100 to 180 V for 1 to 5 hours in NuPAGE MOPS or tris-acetate running buffer, respectively. The protein was then transferred onto a polyvinylidene difluoride (Immobilon F-PL) membrane at 330 mA for 90 min at 4°C. Membranes were blocked for 1 hour in 5% milk in 0.1% PBS–Tween 20 (PBS-T) and then incubated with primary antibodies (see table S5) for 1 hour at room temperature or overnight at 4°C. After three washes in PBS-T, IRdye 680 and 800CW secondary antibodies (1:10,000; LI-COR Biosciences), in PBS-T, were added as appropriate for 1 hour at room temperature. Following a further three washes, membranes were imaged using a LI-COR FC Odyssey CLx. Blot densitometry was analyzed using ImageStudio Lite 5.2 or EmpiriaStudio.

#### RNA immunoprecipitation

Cells were lysed in lysis buffer [50 mM Hepes (pH 7.4), 150 mM NaCl,1% IGEPAL, and 1 mM EDTA] supplemented with RiboLock RNase Inhibitor (0.1 U/μl) and Halt Protease Inhibitor Cocktail and subjected to 10 cycles of sonication (Bioruptor) with alternating 30-s on/off. Lysates were cleared of insoluble material by centrifugation and quantified using DC Assay. One thousand micrograms of the total protein lysate was prepared for each condition. The lysate (10%) was kept aside for total input RNA extraction. Pierce anti-HA magnetic beads (1 mg/ml; Thermo Fisher Scientific) were washed two times with lysis buffer and then added to the protein lysate and rotated for 2 hours at 4°C. Beads were washed four times with lysis buffer and once with PBS each at 4°C followed by elution of RNA in extraction buffer (0.2 M sodium acetate, 1 mM EDTA, and 0.2% SDS), supplemented with RiboLock RNase Inhibitor (0.1 U/μl), for 5 min at 70°C. Total input RNA and immunoprecipitated RNA were purified using the PureLink RNA Micro Scale Kit according to the manufacturer’s instructions with on-column DNase digestion, reverse transcribed using Superscript IV and random hexamers, and then analyzed by qPCR as described.

#### Immunoprecipitation

For SFPQ IP, 0.71 μg of C-terminal SFPQ antibody (Abcam, ab177149) was incubated with prewashed protein G Dynabeads (Invitrogen) in lysis buffer [50 mM tris-HCl (pH 7.4), 150 mM NaCl, 1% IGEPAL + 1 mM EDTA + HALT Protease Inhibitor Cocktail] for 1 hour at 4°C. Eighty to 100 μg of the HEK293T cell lysate, generated by incubating cells with lysis buffer and 10 cycles of sonication (Bioruptor) with alternating 30-s on/off, was added to antibody-conjugated Dynabeads and rotated overnight at 4°C. The input lysate and three times washed beads were boiled at 95°C in 1x LDS buffer + 50 mM DTT. The cleared supernatant was subjected to methanol-chloroform precipitation, before boiling in 1x LDS buffer + 50 mM DTT, to concentrate the protein to enable adequate loading quantity for Western blotting. For HA-SFPQ co-IP, cells were lysed in lysis buffer [50 mM Hepes (pH 7.4), 150 mM NaCl, 1% IGEPAL, and 1 mM EDTA] supplemented with Halt Protease Inhibitor Cocktail and subjected to 10 cycles of sonication (Bioruptor) with alternating 30-s on/off. Lysates were cleared of insoluble material by centrifugation and quantified using DC Assay. One thousand micrograms of the total protein lysate was prepared for each condition. Some lysate was kept aside as an input. Pierce anti-HA magnetic beads (1 mg/ml; Thermo Fisher Scientific) were washed two times with lysis buffer and then added to the protein lysate and rotated overnight at 4°C. The supernatant was kept aside as cleared material. Beads were washed four times with lysis buffer and once with PBS each at 4°C followed by storage at −80°C for MS or boiled at 95°C in 1x LDS buffer + 50 mM DTT and resolved by SDS-PAGE.

#### Immunocytochemistry

HEK293T cells were plated onto poly-d-lysine–coated IBIDI 8-well chamber slides (IBIDI) or 96-well plates (Thermo Fisher Scientific). Before cell staining, cells were fixed using 4% paraformaldehyde for 10 min at room temperature and then washed and maintained in PBS. Fixed cells were permeabilized by 0.3% Triton X-100 in PBS for 10 min and then blocked with 5% bovine serum albumin (BSA) (Sigma-Aldrich) in PBS for 1 hour at room temperature. Immunolabeling was performed with primary antibodies in 5% BSA at 4°C overnight, followed by species-specific Alexa Fluor–conjugated secondary antibodies (1:1000) in 5% BSA for 1 hour at room temperature. 4′,6-Diamidino-2-phenylindole (DAPI) counterstain (100 ng/ml) was subsequently applied for 10 min at room temperature, and then cells were maintained in PBS until imaged. Primary antibodies used are detailed in table S5. Microscopy was carried out using an 880 laser scanning confocal microscope (Zeiss), at 63x objective, 1.4–numerical aperture oil immersion objective, or on an Opera Phenix at 40x objective.

#### BaseScope ISH analysis

Cells cultured on 8-well chamber slides (Thermo Fisher Scientific) were fixed in 10% neutral buffered formalin, dehydrated through increasing concentrations of ethanol, and stored in 100% ethanol at −20°C. On the day of assaying, slides were air-dried, loaded onto the BOND RX (Leica Biosystems), and stained according to the BaseScope LS Reagent Assay protocol. Briefly, samples were rehydrated, subjected to 5-min protease (ACDbio) pretreatment at room temperature to retrieve RNA, and incubated with custom wtSFPQ (TV1) or altSFPQ (TV2) 1xZZ BaseScope probes for 2 hours at 40°C. Probe design and manufacture were carried out by ACDbio/Bio-Techne; probes were designed to span the exon-exon junctions unique to either *wtSFPQ* or *altSFPQ*. The TV1 probe targets NM_005066.3 (transcript variant 1) and should not target NR_136702.2 (transcript variant 2) or NR_136703.2 (transcript variant 3), whereas the TV2 probe targets both NR_136702.2 (tv2) and NR_136703.2 (tv3) and should not target NM_005066.3 (tv1). RNA signal visualization was achieved with AP–Fast Red, and cell nuclei were counterstained with DAPI. Slides were mounted using Vectamount aqueous mourning medium (Vector), and images were acquired on an 880 laser scanning confocal microscope (Zeiss) at 40x objective. Images were initially processed in ImageJ to max project and segment nuclei (StarDist plugin), and then a CellProfiler pipeline was used to identify SFPQ RNA foci using a minimal intensity threshold filter.

#### Statistics

Statistical analysis and associated graph generation were conducted using GraphPad Prism 8. Statistical tests used for each experimental dataset are reported within the figure legends, when significant differences were identified, alongside repeat numbers. When comparing between two sets of data, an unpaired two-tailed Student’s *t* test was used. When comparing between more than two groups, one-way analysis of variance (ANOVA) with Tukey or Dunnett’s post hoc comparison was used, assuming normal distribution. Data are presented as means ± SEM. Statistical significance was accepted at a *P* value of <0.05 and either displayed as actual value or star (*P ≤ 0.05, **P ≤ 0.01, ***P ≤ 0.001, or ****P ≤ 0.0001).

#### RNA-seq analysis

##### RNA-seq processing, integration, and quality control

Raw RNA-seq reads (fastq files) and accompanying metadata were downloaded using the nfcore/fetchngs v1.9 pipeline (*92*) and pysradb v1.3 using the sample sequence read archive accession number. Reads were processed using the nfcore/rnaseq v3.9 pipeline (*92*). Raw reads underwent adaptor trimming with Trim Galore, removal of ribosomal RNA with SortMeRNA, alignment to Ensembl GRCh38.99 human reference genome using splice-aware aligner, STAR v2.7.1, and BAM-level quantification with Salmon. Gene counts were normalized for library size and transformed on a log_2_ scale using the variance stabilizing transformation function in DESeq2. We excluded three control samples in Answer ALS that whole-genome sequencing revealed to have pathogenic ALS mutations. In addition, four Answer ALS iPSMNs from patients with non-ALS motor neuron diseases were excluded. NeuroLINCS consists of three distinct iPSC protocols [induced motor neurons (iMNs), directly induced motor neurons (diMNs), and undifferentiated iPSCs], of which only the iMN and diMN batches were included.

##### Alternative splicing analysis

All modes of alternative splicing were analyzed using MAJIQ v2.4 (*28*, *29*) on iPSMN RNA library samples. STAR-aligned BAMs were used as an input to the MAJIQ splice graph builder using Ensembl GRCh38.99 transcript annotation. For Tyzack *et al.* (*25*), VCP-ALS; Kapeli *et al.* (*55*), FUS-ALS; and Kiskinis *et al.* (*56*), SOD1-ALS datasets, differential splicing was calculated using the MAJIQ deltaPSI function, which is most appropriate for small single-batch homogeneous experimental replicate datasets. For nonmutant sporadic ALS datasets, Answer ALS and NeuroLINCS, differential splicing was calculated using the MAJIQ heterogen function, designed for examining splicing across large and heterogeneous datasets. A threshold of TNOM (thresholded nonparametric overlap measure) *P* value < 0.05 was used to call significant splicing changes between groups. Changes in splicing were examined using the Voila modulize function that breaks down the complex local splice variants into the classic binary splicing events (e.g., exon skipping or IR).

##### Data availability

HiPSC-derived motor neuron raw sequencing data used in this study are available in public repositories under the following accession numbers: Tyzack *et al.* [(*25*); GSE152983], Kapeli *et al.* [(*55*); GSE77702], Kiskinis *et al.* [(*56*); GSE54409], NeuroLINCS Consortium *et al.* [(*57*); phs001231.v2.p1], and Baxi *et al.* [(*58*); Answer ALS data portal]. Some raw data have restricted access (NeuroLINCS dbGaP accession number: phs0001231.v2.p1; Answer ALS database). Granting access to these is beyond the control of the authors. Access can be obtained by applying to the relevant Data Access Committees. Answer ALS requires a signed data use agreement to have full access.

### Mass spectrometry

For on-bead digestion of the pull-down samples, 100 μl of 100 mM triethylammonium bicarbonate (TEAB) was added to the beads followed by simultaneous reduction and alkylation and trypsin digestion with 5 mM tris(2-carboxyethyl)phosphine, 10 mM IAA (indole-3-acetic acid), and a trypsin concentration of 50 ng/μl with overnight shaking at 900 rpm at room temperature. The bead-free digests were SpeedVac dried and reconstituted in 25 μl of 100 mM TEAB, and half of the volume was labeled with the TMTpro reagents by adding 5 μl of the reagent (25 μg/μl). The peptide pool was fractionated with high-pH reversed-phase chromatography using the XBridge C18 column (1.0 mm by 100 mm, 3.5 μm, Waters) on an UltiMate 3000 HPLC system over a 1% gradient in 35 min. Mobile phase A was 0.1% (v/v) ammonium hydroxide, and mobile phase B was 0.1% ammonium hydroxide (v/v) in acetonitrile. Peptide fractions were pooled into eight samples for liquid chromatography–mass spectrometry (LC-MS) analysis. LC-MS analysis was performed on an UltiMate 3000 system coupled with the Orbitrap Ascend mass spectrometer (Thermo Fisher Scientific) using a 25-cm capillary column (Waters, nanoE MZ PST BEH130 C18, 1.7 μm, 75 μm by 250 mm) over a 100-min gradient 5 to 35% of mobile phase B composed of 80% acetonitrile and 0.1% formic acid. Peptides were preconcentrated onto an Acclaim PepMap 100 (100 μm by 2 cm C18, 5 μm) trapping column at 10 μl/min of 0.1% trifluoroacetic acid, and the analytical column was connected to an EASY-Spray emitter (Thermo Fisher Scientific, ES991). MS spectra were collected at an Orbitrap mass resolution of 120k, and precursors were targeted for higher-energy collision dissociation (HCD) fragmentation in the top speed mode (3 s) with a collision energy of 32% and ion trap detection in turbo scan rate. MS3 scans were triggered by Real-Time Search (RTS) against a fasta file containing UniProt *Mus musculus* reviewed canonical and isoform sequences concatenated with the variant sequences of human SFPQ (HA-tag), with multinotch isolation (10 notches) and HCD fragmentation with a collision energy of 55% at 45k Orbitrap resolution. Targeted precursors were dynamically excluded from further activation for 45 s with a mass tolerance of 10 parts per million (ppm), and RTS close-out was disabled. Static modifications for RTS were TMTpro16plex at K/n-term (+304.2071) and carbamidomethyl at C (+57.0215), and variable modifications were deamidated NQ (+0.984) and oxidation of M (+15.9949) with maximum of one missed cleavage and two variable modifications per peptide. The Sequest HT and Comet nodes in Proteome Discoverer 3.0 (Thermo Fisher Scientific) were used to search the raw mass spectra against the same fasta file used in the RTS analysis. The precursor mass tolerance was set at 20 ppm and the fragment ion mass tolerance at 0.5 Da (or 1 Da for Comet) with up to two trypsin missed cleavages allowed. TMTpro at N terminus/K and carbamidomethyl at C were defined as static modifications. Dynamic modifications were oxidation of M and deamidation of N/Q. Peptide confidence was estimated with the Percolator node, and peptide FDR (false discovery rate) was set at 0.01 based on target-decoy search. Only unique peptides were used for quantification, considering protein groups for peptide uniqueness. Peptides with an average reporter signal-to-noise greater than 3 were used for protein quantification.

#### Data availability

The MS proteomics data have been deposited to the ProteomeXchange Consortium via the PRIDE (*93*) partner repository with the dataset identifier PXD051918.

#### LC-MS/MS analysis

Statistical data analysis was performed in the Perseus software (*94*). Normalized protein abundances were exported from Proteome Discoverer and scaled to row mean (log_2_) per replicate batch. Two-sample *t* tests were then performed for WT, ALT, and ΔNLS against the EV samples to identify enriched proteins for each hSFPQ bait protein under both untreated and oxidative stress conditions. In addition, the hSFPQ protein profile was regressed out from the log_2_ scaled data for WT, ALT, and ΔNLS, excluding EVs from the regression; i.e., a linear regression model was applied, using hSFPQ relative abundances as the independent variable to normalize target protein abundances, assuming a linear relationship between bait and prey signals. The hSFPQ scaled log_2_ values were used as the independent variable (*x*), and the log_2_ scaled values of each protein were used as the dependent variable (*y*). The residuals of the *y* = *ax* + *b* (*a* is the slope, and *b* is the intercept) model for each protein were used as the corrected relative log_2_ ratios. Pearson correlation values for each protein to hSFPQ were also computed. Matched log_2_ ratios comparing ALT and ΔNLS against WT as well as ΔNLS versus ALT were further computed followed by one-sample *t* tests to identify differences between the hSFPQ conditions. Last, π values (log_2_FC × −log_10_*P* value) were computed (*95*) only for proteins with positive correlation to hSFPQ (Pearson > 0.3) as a means of significance ranking. The ranking scores were used for network cluster enrichment in STRING (*54*).

## References

[1] M. Neumann, D. M. Sampathu, L. K. Kwong, A. C. Truax, M. C. Micsenyi, T. T. Chou, J. Bruce, T. Schuck, M. Grossman, C. M. Clark, L. F. McCluskey, B. L. Miller, E. Masliah, I. R. Mackenzie, H. Feldman, W. Feiden, H. A. Kretzschmar, J. Q. Trojanowski, V. M. Lee, Ubiquitinated TDP-43 in frontotemporal lobar degeneration and amyotrophic lateral sclerosis. Science 314, 130–133 (2006). 17023659 10.1126/science.1134108

[2] T. J. Kwiatkowski Jr., D. A. Bosco, A. L. Leclerc, E. Tamrazian, C. R. Vanderburg, C. Russ, A. Davis, J. Gilchrist, E. J. Kasarskis, T. Munsat, P. Valdmanis, G. A. Rouleau, B. A. Hosler, P. Cortelli, P. J. de Jong, Y. Yoshinaga, J. L. Haines, M. A. Pericak-Vance, J. Yan, N. Ticozzi, T. Siddique, D. McKenna-Yasek, P. C. Sapp, H. R. Horvitz, J. E. Landers, R. H. Brown Jr., Mutations in the FUS/TLS gene on chromosome 16 cause familial amyotrophic lateral sclerosis. Science 323, 1205–1208 (2009). 19251627 10.1126/science.1166066

[3] C. Vance, B. Rogelj, T. Hortobágyi, K. J. De Vos, A. L. Nishimura, J. Sreedharan, X. Hu, B. Smith, D. Ruddy, P. Wright, J. Ganesalingam, K. L. Williams, V. Tripathi, S. Al-Saraj, A. Al-Chalabi, P. N. Leigh, I. P. Blair, G. Nicholson, J. de Belleroche, J.-M. Gallo, C. C. Miller, C. E. Shaw, Mutations in FUS, an RNA processing protein, cause familial amyotrophic lateral sclerosis type 6. Science 323, 1208–1211 (2009). 19251628 10.1126/science.1165942 PMC4516382

[4] G. E. Tyzack, R. Luisier, D. M. Taha, J. Neeves, M. Modic, J. S. Mitchell, I. Meyer, L. Greensmith, J. Newcombe, J. Ule, N. M. Luscombe, R. Patani, Widespread FUS mislocalization is a molecular hallmark of amyotrophic lateral sclerosis. Brain 142, 2572–2580 (2019). 31368485 10.1093/brain/awz217 PMC6735815

[5] R. Luisier, G. E. Tyzack, C. E. Hall, J. S. Mitchell, H. Devine, D. M. Taha, B. Malik, I. Meyer, L. Greensmith, J. Newcombe, J. Ule, N. M. Luscombe, R. Patani, Intron retention and nuclear loss of SFPQ are molecular hallmarks of ALS. Nat. Commun. 9, 2010 (2018). 29789581 10.1038/s41467-018-04373-8 PMC5964114

[6] C. A. Yarosh, J. R. Iacona, C. S. Lutz, K. W. Lynch, PSF: Nuclear busy-body or nuclear facilitator? Wiley Interdiscip. Rev. RNA 6, 351–367 (2015). 25832716 10.1002/wrna.1280 PMC4478221

[7] S. Roepcke, S. Stahlberg, H. Klein, M. H. Schulz, L. Theobald, S. Gohlke, M. Vingron, D. J. Walther, A tandem sequence motif acts as a distance-dependent enhancer in a set of genes involved in translation by binding the proteins NonO and SFPQ. BMC Genomics 12, 624 (2011). 22185324 10.1186/1471-2164-12-624 PMC3262029

[8] F. Heyd, K. W. Lynch, Phosphorylation-dependent regulation of PSF by GSK3 controls CD45 alternative splicing. Mol. Cell 40, 126–137 (2010). 20932480 10.1016/j.molcel.2010.09.013 PMC2954053

[9] A. H. Fox, Y. W. Lam, A. K. L. Leung, C. E. Lyon, J. Andersen, M. Mann, A. I. Lamond, Paraspeckles: A novel nuclear domain. Curr. Biol. 12, 13–25 (2002). 11790299 10.1016/s0960-9822(01)00632-7

[10] P. M. Gordon, F. Hamid, E. V. Makeyev, C. Houart, A conserved role for the ALS-linked splicing factor SFPQ in repression of pathogenic cryptic last exons. Nat. Commun. 12, 1918 (2021). 33771997 10.1038/s41467-021-22098-z PMC7997972

[11] A. Takeuchi, K. Iida, T. Tsubota, M. Hosokawa, M. Denawa, J. B. Brown, K. Ninomiya, M. Ito, H. Kimura, T. Abe, H. Kiyonari, K. Ohno, M. Hagiwara, Loss of Sfpq causes long-gene transcriptopathy in the brain. Cell Rep. 23, 1326–1341 (2018). 29719248 10.1016/j.celrep.2018.03.141

[12] S. Ishigaki, Y. Fujioka, Y. Okada, Y. Riku, T. Udagawa, D. Honda, S. Yokoi, K. Endo, K. Ikenaka, S. Takagi, Y. Iguchi, N. Sahara, A. Takashima, H. Okano, M. Yoshida, H. Warita, M. Aoki, H. Watanabe, H. Okado, M. Katsuno, G. Sobue, Altered tau isoform ratio caused by loss of FUS and SFPQ function leads to FTLD-like phenotypes. Cell Rep. 18, 1118–1131 (2017). 28147269 10.1016/j.celrep.2017.01.013

[13] C. Hung, R. Patani, Elevated 4R tau contributes to endolysosomal dysfunction and neurodegeneration in VCP-related frontotemporal dementia. Brain 147, 970–979 (2024). 37882537 10.1093/brain/awad370 PMC10907086

[14] Y. Kanai, N. Dohmae, N. Hirokawa, Kinesin transports RNA: Isolation and characterization of an RNA-transporting granule. Neuron 43, 513–525 (2004). 15312650 10.1016/j.neuron.2004.07.022

[15] M. T. Furukawa, H. Sakamoto, K. Inoue, Interaction and colocalization of HERMES/RBPMS with NonO, PSF, and G3BP1 in neuronal cytoplasmic RNP granules in mouse retinal line cells. Genes Cells 20, 257–266 (2015). 25651939 10.1111/gtc.12224

[16] K. E. Cosker, S. J. Fenstermacher, M. F. Pazyra-Murphy, H. L. Elliott, R. A. Segal, The RNA-binding protein SFPQ orchestrates an RNA regulon to promote axon viability. Nat. Neurosci. 19, 690–696 (2016). 27019013 10.1038/nn.4280 PMC5505173

[17] S. Thomas-Jinu, P. M. Gordon, T. Fielding, R. Taylor, B. N. Smith, V. Snowden, E. Blanc, C. Vance, S. Topp, C.-H. Wong, H. Bielen, K. L. Williams, E. P. McCann, G. A. Nicholson, A. Pan-Vazquez, A. H. Fox, C. S. Bond, W. S. Talbot, I. P. Blair, C. E. Shaw, C. Houart, Non-nuclear pool of splicing factor SFPQ regulates axonal transcripts required for normal motor development. Neuron 94, 322–336.e5 (2017). 28392072 10.1016/j.neuron.2017.03.026 PMC5405110

[18] Y. D. Ke, J. Dramiga, U. Schütz, J. J. Kril, L. M. Ittner, H. Schröder, J. Götz, Tau-mediated nuclear depletion and cytoplasmic accumulation of SFPQ in Alzheimer’s and Pick’s disease. PLOS ONE 7, e35678 (2012). 22558197 10.1371/journal.pone.0035678 PMC3338448

[19] J. Widagdo, S. Udagedara, N. Bhembre, J. Z. A. Tan, L. Neureiter, J. Huang, V. Anggono, M. Lee, Familial ALS-associated *SFPQ* variants promote the formation of SFPQ cytoplasmic aggregates in primary neurons. Open Biol. 12, 220187 (2022). 36168806 10.1098/rsob.220187 PMC9516340

[20] N. Younas, S. Zafar, M. Shafiq, A. Noor, A. Siegert, A. S. Arora, A. Galkin, A. Zafar, M. Schmitz, C. Stadelmann, O. Andreoletti, I. Ferrer, I. Zerr, SFPQ and Tau: Critical factors contributing to rapid progression of Alzheimer’s disease. Acta Neuropathol. 140, 317–339 (2020). 32577828 10.1007/s00401-020-02178-y PMC7423812

[21] J. Lu, R. Shu, Y. Zhu, Dysregulation and dislocation of SFPQ disturbed DNA organization in Alzheimer’s disease and frontotemporal dementia. J. Alzheimers Dis. 61, 1311–1321 (2018). 29376859 10.3233/JAD-170659

[22] J. Huang, M. Ringuet, A. E. Whitten, S. Caria, Y. W. Lim, R. Badhan, V. Anggono, M. Lee, Structural basis of the zinc-induced cytoplasmic aggregation of the RNA-binding protein SFPQ. Nucleic Acids Res. 48, 3356–3365 (2020). 32034402 10.1093/nar/gkaa076 PMC7102971

[23] G. E. Tyzack, G. Manferrari, J. Newcombe, N. M. Luscombe, R. Luisier, R. Patani, Paraspeckle components NONO and PSPC1 are not mislocalized from motor neuron nuclei in sporadic ALS. Brain 143, e66 (2020). 32844195 10.1093/brain/awaa205 PMC7447511

[24] A. L. Hogan, N. Grima, J. A. Fifita, E. P. McCann, B. Heng, S. C. M. Fat, S. Wu, R. Maharjan, A. K. Cain, L. Henden, S. Rayner, I. Tarr, K. Y. Zhang, Q. Zhao, Z.-H. Zhang, A. Wright, A. Lee, M. Morsch, S. Yang, K. L. Williams, I. P. Blair, Splicing factor proline and glutamine rich intron retention, reduced expression and aggregate formation are pathological features of amyotrophic lateral sclerosis. Neuropathol. Appl. Neurobiol. 47, 990–1003 (2021). 34288034 10.1111/nan.12749

[25] G. E. Tyzack, J. Neeves, H. Crerar, P. Klein, O. Ziff, D. M. Taha, R. Luisier, N. M. Luscombe, R. Patani, Aberrant cytoplasmic intron retention is a blueprint for RNA binding protein mislocalization in VCP-related amyotrophic lateral sclerosis. Brain 144, 1985–1993 (2021). 33693641 10.1093/brain/awab078 PMC8370440

[26] J. G. Patton, E. B. Porro, J. Galceran, P. Tempst, B. Nadal-Ginard, Cloning and characterization of PSF, a novel pre-mRNA splicing factor. Genes Dev. 7, 393–406 (1993). 8449401 10.1101/gad.7.3.393

[27] P. Kim, M. Yang, K. Yiya, W. Zhao, X. Zhou, ExonSkipDB: Functional annotation of exon skipping event in human. Nucleic Acids Res. 48, D896–D907 (2020). 31642488 10.1093/nar/gkz917 PMC7145592

[28] J. Vaquero-Garcia, A. Barrera, M. R. Gazzara, J. Gonzalez-Vallinas, N. F. Lahens, J. B. Hogenesch, K. W. Lynch, Y. Barash, A new view of transcriptome complexity and regulation through the lens of local splicing variations. eLife 5, e11752 (2016). 26829591 10.7554/eLife.11752 PMC4801060

[29] J. Vaquero-Garcia, J. K. Aicher, S. Jewell, M. R. Gazzara, C. M. Radens, A. Jha, S. S. Norton, N. F. Lahens, G. R. Grant, Y. Barash, RNA splicing analysis using heterogeneous and large RNA-seq datasets. Nat. Commun. 14, 1230 (2023). 36869033 10.1038/s41467-023-36585-y PMC9984406

[30] V. T. Nguyen, F. Giannoni, M. F. Dubois, S. J. Seo, M. Vigneron, C. Kédinger, O. Bensaude, In vivo degradation of RNA polymerase II largest subunit triggered by alpha-amanitin. Nucleic Acids Res. 24, 2924–2929 (1996). 8760875 10.1093/nar/24.15.2924 PMC146057

[31] D. A. Bushnell, P. Cramer, R. D. Kornberg, Structural basis of transcription: α-amanitin-RNA polymerase II cocrystal at 2.8 Å resolution. Proc. Natl. Acad. Sci. U.S.A. 99, 1218–1222 (2002). 11805306 10.1073/pnas.251664698 PMC122170

[32] E. Nagy, L. E. Maquat, A rule for termination-codon position within intron-containing genes: When nonsense affects RNA abundance. Trends Biochem. Sci. 23, 198–199 (1998). 9644970 10.1016/s0968-0004(98)01208-0

[33] F. Lejeune, L. E. Maquat, Mechanistic links between nonsense-mediated mRNA decay and pre-mRNA splicing in mammalian cells. Curr. Opin. Cell Biol. 17, 309–315 (2005). 15901502 10.1016/j.ceb.2005.03.002

[34] D. A. Santos, L. Shi, B. P. Tu, J. S. Weissman, Cycloheximide can distort measurements of mRNA levels and translation efficiency. Nucleic Acids Res. 47, 4974–4985 (2019). 30916348 10.1093/nar/gkz205 PMC6547433

[35] L. M. Langer, F. Bonneau, Y. Gat, E. Conti, Cryo-EM reconstructions of inhibitor-bound SMG1 kinase reveal an autoinhibitory state dependent on SMG8. Elife 10, e72353 (2021). 34698635 10.7554/eLife.72353 PMC8592573

[36] L. F. Lareau, A. N. Brooks, D. A. W. Soergel, Q. Meng, S. E. Brenner, The coupling of alternative splicing and nonsense-mediated mRNA decay. Adv. Exp. Med. Biol. 623, 190–211 (2007). 18380348 10.1007/978-0-387-77374-2_12

[37] J. Z. Ni, L. Grate, J. P. Donohue, C. Preston, N. Nobida, G. O’Brien, L. Shiue, T. A. Clark, J. E. Blume, M. Ares Jr., Ultraconserved elements are associated with homeostatic control of splicing regulators by alternative splicing and nonsense-mediated decay. Genes Dev. 21, 708–718 (2007). 17369403 10.1101/gad.1525507 PMC1820944

[38] S. Campagne, D. Jutzi, F. Malard, M. Matoga, K. Romane, M. Feldmuller, M. Colombo, M.-D. Ruepp, F. H.-T. Allain, Molecular basis of RNA-binding and autoregulation by the cancer-associated splicing factor RBM39. Nat. Commun. 14, 5366 (2023). 37666821 10.1038/s41467-023-40254-5 PMC10477243

[39] J. P. B. Lloyd, C. E. French, S. E. Brenner, Polysome fractionation analysis reveals features important for human nonsense-mediated mRNA decay. bioRxiv 981811 [Preprint] (2020). 10.1101/2020.03.08.981811.

[40] L. E. Maquat, W.-Y. Tarn, O. Isken, The pioneer round of translation: Features and functions. Cell 142, 368–374 (2010). 20691898 10.1016/j.cell.2010.07.022 PMC2950652

[41] O. Isken, Y. K. Kim, N. Hosoda, G. L. Mayeur, J. W. Hershey, L. E. Maquat, Upf1 phosphorylation triggers translational repression during nonsense-mediated mRNA decay. Cell 133, 314–327 (2008). 18423202 10.1016/j.cell.2008.02.030 PMC4193665

[42] W. K. Kim, S. Yun, Y. Kwon, K. T. You, N. Shin, J. Kim, H. Kim, mRNAs containing NMD-competent premature termination codons are stabilized and translated under UPF1 depletion. Sci. Rep. 7, 15833 (2017). 29158530 10.1038/s41598-017-16177-9 PMC5696521

[43] B. T. Dye, J. G. Patton, An RNA recognition motif (RRM) is required for the localization of PTB-associated splicing factor (PSF) to subnuclear speckles. Exp. Cell Res. 263, 131–144 (2001). 11161712 10.1006/excr.2000.5097

[44] G. J. Knott, Y. S. Chong, D. M. Passon, X.-H. Liang, E. Deplazes, M. R. Conte, A. C. Marshall, M. Lee, A. H. Fox, C. S. Bond, Structural basis of dimerization and nucleic acid binding of human DBHS proteins NONO and PSPC1. Nucleic Acids Res. 50, 522–535 (2022). 34904671 10.1093/nar/gkab1216 PMC8754649

[45] J. Huang, G. P. Casas Garcia, M. A. Perugini, A. H. Fox, C. S. Bond, M. Lee, Crystal structure of a SFPQ/PSPC1 heterodimer provides insights into preferential heterodimerization of human DBHS family proteins. J. Biol. Chem. 293, 6593–6602 (2018). 29530979 10.1074/jbc.RA117.001451 PMC5925804

[46] A. C. Marshall, J. Cummins, S. Kobelke, T. Zhu, J. Widagdo, V. Anggono, A. Hyman, A. H. Fox, C. S. Bond, M. Lee, Different low-complexity regions of SFPQ play distinct roles in the formation of biomolecular condensates. J. Mol. Biol. 435, 168364 (2023). 37952770 10.1016/j.jmb.2023.168364

[47] J. Lee, H. Cho, I. Kwon, Phase separation of low-complexity domains in cellular function and disease. Exp. Mol. Med. 54, 1412–1422 (2022). 36175485 10.1038/s12276-022-00857-2 PMC9534829

[48] T. Naganuma, S. Nakagawa, A. Tanigawa, Y. F. Sasaki, N. Goshima, T. Hirose, Alternative 3′-end processing of long noncoding RNA initiates construction of nuclear paraspeckles. EMBO J. 31, 4020–4034 (2012). 22960638 10.1038/emboj.2012.251 PMC3474925

[49] J. Harley, R. Patani, Stress-specific spatiotemporal responses of RNA-binding proteins in human stem-cell-derived motor neurons. Int. J. Mol. Sci. 21, 8346 (2020). 33172210 10.3390/ijms21218346 PMC7664327

[50] P. L. Boutz, A. Bhutkar, P. A. Sharp, Detained introns are a novel, widespread class of post-transcriptionally spliced introns. Genes Dev. 29, 63–80 (2015). 25561496 10.1101/gad.247361.114 PMC4281565

[51] S.-K. Park, X. Zhou, K. E. Pendleton, O. V. Hunter, J. J. Kohler, K. A. O’Donnell, N. K. Conrad, A conserved splicing silencer dynamically regulates O-GlcNAc transferase intron retention and O-GlcNAc homeostasis. Cell Rep. 20, 1088–1099 (2017). 28768194 10.1016/j.celrep.2017.07.017 PMC5588854

[52] Z.-W. Tan, G. Fei, J. A. Paulo, S. Bellaousov, S. E. S. Martin, D. Y. Duveau, C. J. Thomas, S. P. Gygi, P. L. Boutz, S. Walker, O-GlcNAc regulates gene expression by controlling detained intron splicing. Nucleic Acids Res. 48, 5656–5669 (2020). 32329777 10.1093/nar/gkaa263 PMC7261177

[53] T. Yamazaki, S. Souquere, T. Chujo, S. Kobelke, Y. S. Chong, A. H. Fox, C. S. Bond, S. Nakagawa, G. Pierron, T. Hirose, Functional domains of NEAT1 architectural lncRNA induce paraspeckle assembly through phase separation. Mol. Cell 70, 1038–1053.e7 (2018). 29932899 10.1016/j.molcel.2018.05.019

[54] D. Szklarczyk, J. H. Morris, H. Cook, M. Kuhn, S. Wyder, M. Simonovic, A. Santos, N. T. Doncheva, A. Roth, P. Bork, L. J. Jensen, C. von Mering, The STRING database in 2017: Quality-controlled protein-protein association networks, made broadly accessible. Nucleic Acids Res. 45, D362–D368 (2017). 27924014 10.1093/nar/gkw937 PMC5210637

[55] K. Kapeli, G. A. Pratt, A. Q. Vu, K. R. Hutt, F. J. Martinez, B. Sundararaman, R. Batra, P. Freese, N. J. Lambert, S. C. Huelga, S. J. Chun, T. Y. Liang, J. Chang, J. P. Donohue, L. Shiue, J. Zhang, H. Zhu, F. Cambi, E. Kasarskis, S. Hoon, M. Ares Jr., C. B. Burge, J. Ravits, F. Rigo, G. W. Yeo, Distinct and shared functions of ALS-associated proteins TDP-43, FUS and TAF15 revealed by multisystem analyses. Nat. Commun. 7, 12143 (2016). 27378374 10.1038/ncomms12143 PMC4935974

[56] E. Kiskinis, J. Sandoe, L. A. Williams, G. L. Boulting, R. Moccia, B. J. Wainger, S. Han, T. Peng, S. Thams, S. Mikkilineni, C. Mellin, F. T. Merkle, B. N. Davis-Dusenbery, M. Ziller, D. Oakley, J. Ichida, S. D. Costanzo, N. Atwater, M. L. Maeder, M. J. Goodwin, J. Nemesh, R. E. Handsaker, D. Paull, S. Noggle, S. A. McCarroll, J. K. Joung, C. J. Woolf, R. H. Brown, K. Eggan, Pathways disrupted in human ALS motor neurons identified through genetic correction of mutant SOD1. Cell Stem Cell 14, 781–795 (2014). 24704492 10.1016/j.stem.2014.03.004 PMC4653065

[57] NeuroLINCS Consortium, J. Li, R. G. Lim, J. A. Kaye, V. Dardov, A. N. Coyne, J. Wu, P. Milani, A. Cheng, T. G. Thompson, L. Ornelas, A. Frank, M. Adam, M. G. Banuelos, M. Casale, V. Cox, R. Escalante-Chong, J. G. Daigle, E. Gomez, L. Hayes, R. Holewenski, S. Lei, A. Lenail, L. Lima, B. Mandefro, A. Matlock, L. Panther, N. L. Patel-Murray, J. Pham, D. Ramamoorthy, K. Sachs, B. Shelley, J. Stocksdale, H. Trost, M. Wilhelm, V. Venkatraman, B. T. Wassie, S. Wyman, S. Yang, NYGC ALS Consortium, J. E. Van Eyk, T. E. Lloyd, S. Finkbeiner, E. Fraenkel, J. D. Rothstein, D. Sareen, C. N. Svendsen, L. M. Thompson, An integrated multi-omic analysis of iPSC-derived motor neurons from C9ORF72 ALS patients. iScience 24, 103221 (2021). 34746695 10.1016/j.isci.2021.103221 PMC8554488

[58] E. G. Baxi, T. Thompson, J. Li, J. A. Kaye, R. G. Lim, J. Wu, D. Ramamoorthy, L. Lima, V. Vaibhav, A. Matlock, A. Frank, A. N. Coyne, B. Landin, L. Ornelas, E. Mosmiller, S. Thrower, S. M. Farr, L. Panther, E. Gomez, E. Galvez, D. Perez, I. Meepe, S. Lei, B. Mandefro, H. Trost, L. Pinedo, M. G. Banuelos, C. Liu, R. Moran, V. Garcia, M. Workman, R. Ho, S. Wyman, J. Roggenbuck, M. B. Harms, J. Stocksdale, R. Miramontes, K. Wang, V. Venkatraman, R. Holewenski, N. Sundararaman, R. Pandey, D.-M. Manalo, A. Donde, N. Huynh, M. Adam, B. T. Wassie, E. Vertudes, N. Amirani, K. Raja, R. Thomas, L. Hayes, A. Lenail, A. Cerezo, S. Luppino, A. Farrar, L. Pothier, C. Prina, T. Morgan, A. Jamil, S. Heintzman, J. Jockel-Balsarotti, E. Karanja, J. Markway, M. M. Callum, B. Joslin, D. Alibazoglu, S. Kolb, S. Ajroud-Driss, R. Baloh, D. Heitzman, T. Miller, J. D. Glass, N. L. Patel-Murray, H. Yu, E. Sinani, P. Vigneswaran, A. V. Sherman, O. Ahmad, P. Roy, J. C. Beavers, S. Zeiler, J. W. Krakauer, C. Agurto, G. Cecchi, M. Bellard, Y. Raghav, K. Sachs, T. Ehrenberger, E. Bruce, M. E. Cudkowicz, N. Maragakis, R. Norel, J. E. Van Eyk, S. Finkbeiner, J. Berry, D. Sareen, L. M. Thompson, E. Fraenkel, C. N. Svendsen, J. D. Rothstein, Answer ALS, a large-scale resource for sporadic and familial ALS combining clinical and multi-omics data from induced pluripotent cell lines. Nat. Neurosci. 25, 226–237 (2022). 35115730 10.1038/s41593-021-01006-0 PMC8825283

[59] C. Dingwall, R. A. Laskey, Nuclear targeting sequences—A consensus? Trends Biochem. Sci. 16, 478–481 (1991). 1664152 10.1016/0968-0004(91)90184-w

[60] K. Weskamp, E. M. Tank, R. Miguez, J. P. McBride, N. B. Gómez, M. White, Z. Lin, C. M. Gonzalez, A. Serio, J. Sreedharan, S. J. Barmada, Shortened TDP43 isoforms upregulated by neuronal hyperactivity drive TDP43 pathology in ALS. J. Clin. Invest. 130, 1139–1155 (2020). 31714900 10.1172/JCI130988 PMC7269575

[61] M. Shenouda, S. Xiao, L. MacNair, A. Lau, J. Robertson, A C-terminally truncated TDP-43 splice isoform exhibits neuronal specific cytoplasmic aggregation and contributes to TDP-43 pathology in ALS. Front. Neurosci. 16, 868556 (2022). 35801182 10.3389/fnins.2022.868556 PMC9253772

[62] Y. Zeng, A. Lovchykova, T. Akiyama, C. Liu, C. Guo, V. M. Jawahar, O. Sianto, A. Calliari, M. Prudencio, D. W. Dickson, L. Petrucelli, A. D. Gitler, TDP-43 nuclear loss in FTD/ALS causes widespread alternative polyadenylation changes. bioRxiv 576625 [Preprint] (2024). 10.1101/2024.01.22.575730. PMC12586192 41120750

[63] J. G. Patton, S. A. Mayer, P. Tempst, B. Nadal-Ginard, Characterization and molecular cloning of polypyrimidine tract-binding protein: A component of a complex necessary for pre-mRNA splicing. Genes Dev. 5, 1237–1251 (1991). 1906036 10.1101/gad.5.7.1237

[64] D. Flather, J. H. C. Nguyen, B. L. Semler, P. D. Gershon, Exploitation of nuclear functions by human rhinovirus, a cytoplasmic RNA virus. PLOS Pathog. 14, e1007277 (2018). 30142213 10.1371/journal.ppat.1007277 PMC6126879

[65] G. J. Knott, C. S. Bond, A. H. Fox, The DBHS proteins SFPQ, NONO and PSPC1: A multipurpose molecular scaffold. Nucleic Acids Res. 44, 3989–4004 (2016). 27084935 10.1093/nar/gkw271 PMC4872119

[66] Y. Fukuda, M. F. Pazyra-Murphy, E. S. Silagi, O. E. Tasdemir-Yilmaz, Y. Li, L. Rose, Z. C. Yeoh, N. E. Vangos, E. A. Geffken, H.-S. Seo, G. Adelmant, G. H. Bird, L. D. Walensky, J. A. Marto, S. Dhe-Paganon, R. A. Segal, Binding and transport of SFPQ-RNA granules by KIF5A/KLC1 motors promotes axon survival. J. Cell Biol. 220, e202005051 (2021). 33284322 10.1083/jcb.202005051 PMC7721913

[67] M. Lee, A. Sadowska, I. Bekere, D. Ho, B. S. Gully, Y. Lu, K. S. Iyer, J. Trewhella, A. H. Fox, C. S. Bond, The structure of human SFPQ reveals a coiled-coil mediated polymer essential for functional aggregation in gene regulation. Nucleic Acids Res. 43, 3826–3840 (2015). 25765647 10.1093/nar/gkv156 PMC4402515

[68] J. M. Taliaferro, M. Vidaki, R. Oliveira, S. Olson, L. Zhan, T. Saxena, E. T. Wang, B. R. Graveley, F. B. Gertler, M. S. Swanson, C. B. Burge, Distal alternative last exons localize mRNAs to neural projections. Mol. Cell 61, 821–833 (2016). 26907613 10.1016/j.molcel.2016.01.020 PMC4798900

[69] T. Shigeoka, H. Jung, J. Jung, B. Turner-Bridger, J. Ohk, J. Q. Lin, P. S. Amieux, C. E. Holt, Dynamic axonal translation in developing and mature visual circuits. Cell 166, 181–192 (2016). 27321671 10.1016/j.cell.2016.05.029 PMC4930487

[70] E. A. Lysikova, S. Funikov, A. P. Rezvykh, K. D. Chaprov, M. S. Kukharsky, A. Ustyugov, A. V. Deykin, I. M. Flyamer, S. Boyle, S. O. Bachurin, N. Ninkina, V. L. Buchman, Low level of expression of C-terminally truncated human FUS causes extensive changes in the spinal cord transcriptome of asymptomatic transgenic mice. Neurochem. Res. 45, 1168–1179 (2020). 32157564 10.1007/s11064-020-02999-z

[71] S. Reber, J. Mechtersheimer, S. Nasif, J. A. Benitez, M. Colombo, M. Domanski, D. Jutzi, E. Hedlund, M. D. Ruepp, CRISPR-Trap: A clean approach for the generation of gene knockouts and gene replacements in human cells. Mol. Biol. Cell 29, 75–83 (2018). 29167381 10.1091/mbc.E17-05-0288 PMC5909934

[72] C. Batlle, P. Yang, M. Coughlin, J. Messing, M. Pesarrodona, E. Szulc, X. Salvatella, H. J. Kim, J. P. Taylor, S. Ventura, hnRNPDL phase separation is regulated by alternative splicing and disease-causing mutations accelerate its aggregation. Cell Rep. 30, 1117–1128.e5 (2020). 31995753 10.1016/j.celrep.2019.12.080 PMC6996132

[73] A. Patel, H. O. Lee, L. Jawerth, S. Maharana, M. Jahnel, M. Y. Hein, S. Stoynov, J. Mahamid, S. Saha, T. M. Franzmann, A. Pozniakovski, I. Poser, N. Maghelli, L. A. Royer, M. Weigert, E. W. Myers, S. Grill, D. Drechsel, A. A. Hyman, S. Alberti, A liquid-to-solid phase transition of the ALS protein FUS accelerated by disease mutation. Cell 162, 1066–1077 (2015). 26317470 10.1016/j.cell.2015.07.047

[74] S. Reber, D. Jutzi, H. Lindsay, A. Devoy, J. Mechtersheimer, B. R. Levone, M. Domanski, E. Bentmann, D. Dormann, O. Muhlemann, S. M. L. Barabino, M.-D. Ruepp, The phase separation-dependent FUS interactome reveals nuclear and cytoplasmic function of liquid-liquid phase separation. Nucleic Acids Res. 49, 7713–7731 (2021). 34233002 10.1093/nar/gkab582 PMC8287939

[75] S. Alberti, A. Gladfelter, T. Mittag, Considerations and challenges in studying liquid-liquid phase separation and biomolecular condensates. Cell 176, 419–434 (2019). 30682370 10.1016/j.cell.2018.12.035 PMC6445271

[76] A. Molliex, J. Temirov, J. Lee, M. Coughlin, A. P. Kanagaraj, H. J. Kim, T. Mittag, J. P. Taylor, Phase separation by low complexity domains promotes stress granule assembly and drives pathological fibrillization. Cell 163, 123–133 (2015). 26406374 10.1016/j.cell.2015.09.015 PMC5149108

[77] D. Simsek, G. C. Tiu, R. A. Flynn, G. W. Byeon, K. Leppek, A. F. Xu, H. Y. Chang, M. Barna, The mammalian ribo-interactome reveals ribosome functional diversity and heterogeneity. Cell 169, 1051–1065.e18 (2017). 28575669 10.1016/j.cell.2017.05.022 PMC5548193

[78] S. Sun, Y. Sun, S. C. Ling, L. Ferraiuolo, M. McAlonis-Downes, Y. Zou, K. Drenner, Y. Wang, D. Ditsworth, S. Tokunaga, A. Kopelevich, B. K. Kaspar, C. Lagier-Tourenne, D. W. Cleveland, Translational profiling identifies a cascade of damage initiated in motor neurons and spreading to glia in mutant SOD1-mediated ALS. Proc. Natl. Acad. Sci. U.S.A. 112, E6993–E7002 (2015). 26621731 10.1073/pnas.1520639112 PMC4687558

[79] E. Kiesler, F. Miralles, A.-K. Ostlund Farrants, N. Visa, The Hrp65 self-interaction is mediated by an evolutionarily conserved domain and is required for nuclear import of Hrp65 isoforms that lack a nuclear localization signal. J. Cell Sci. 116, 3949–3956 (2003). 12928329 10.1242/jcs.00690

[80] S. Zhang, J. A. Cooper, Y. S. Chong, A. Naveed, C. Mayoh, N. Jayatilleke, T. Liu, S. Amos, S. Kobelke, A. C. Marshall, O. Meers, Y. S. Choi, C. S. Bond, A. H. Fox, NONO enhances mRNA processing of super-enhancer-associated GATA2 and HAND2 genes in neuroblastoma. EMBO Rep. 24, e54977 (2023). 36416237 10.15252/embr.202254977 PMC9900351

[81] W. Shao, X. Bi, Y. Pan, B. Gao, J. Wu, Y. Yin, Z. Liu, M. Peng, W. Zhang, X. Jiang, W. Ren, Y. Xu, Z. Wu, K. Wang, G. Zhan, J. Y. Lu, X. Han, T. Li, J. Wang, G. Li, H. Deng, B. Li, X. Shen, Phase separation of RNA-binding protein promotes polymerase binding and transcription. Nat. Chem. Biol. 18, 70–80 (2022). 34916619 10.1038/s41589-021-00904-5

[82] S. Maharana, J. Wang, D. K. Papadopoulos, D. Richter, A. Pozniakovsky, I. Poser, M. Bickle, S. Rizk, J. Guillén-Boixet, T. M. Franzmann, M. Jahnel, L. Marrone, Y.-T. Chang, J. Sterneckert, P. Tomancak, A. A. Hyman, S. Alberti, RNA buffers the phase separation behavior of prion-like RNA binding proteins. Science 360, 918–921 (2018). 29650702 10.1126/science.aar7366 PMC6091854

[83] K. Ninomiya, N. Kataoka, M. Hagiwara, Stress-responsive maturation of Clk1/4 pre-mRNAs promotes phosphorylation of SR splicing factor. J. Cell Biol. 195, 27–40 (2011). 21949414 10.1083/jcb.201107093 PMC3187705

[84] C. Naro, A. Jolly, S. D. Persio, P. Bielli, N. Setterblad, A. J. Alberdi, E. Vicini, R. Geremia, P. De la Grange, C. Sette, An orchestrated intron retention program in meiosis controls timely usage of transcripts during germ cell differentiation. Dev. Cell 41, 82–93.e4 (2017). 28366282 10.1016/j.devcel.2017.03.003 PMC5392497

[85] D. Y. Vargas, K. Shah, M. Batish, M. Levandoski, S. Sinha, S. A. Marras, P. Schedl, S. Tyagi, Single-molecule imaging of transcriptionally coupled and uncoupled splicing. Cell 147, 1054–1065 (2011). 22118462 10.1016/j.cell.2011.10.024 PMC3245879

[86] A. A. Pai, T. Henriques, K. McCue, A. Burkholder, K. Adelman, C. B. Burge, The kinetics of pre-mRNA splicing in the Drosophila genome and the influence of gene architecture. Elife 6, e32537 (2017). 29280736 10.7554/eLife.32537 PMC5762160

[87] R. M. Martin, J. Rino, C. Carvalho, T. Kirchhausen, M. Carmo-Fonseca, Live-cell visualization of pre-mRNA splicing with single-molecule sensitivity. Cell Rep. 4, 1144–1155 (2013). 24035393 10.1016/j.celrep.2013.08.013 PMC3805459

[88] C. E. Hall, Z. Yao, M. Choi, G. E. Tyzack, A. Serio, R. Luisier, J. Harley, E. Preza, C. Arber, S. J. Crisp, P. M. D. Watson, D. M. Kullmann, A. Y. Abramov, S. Wray, R. Burley, S. H. Y. Loh, L. M. Martins, M. M. Stevens, N. M. Luscombe, C. R. Sibley, A. Lakatos, J. Ule, S. Gandhi, R. Patani, Progressive motor neuron pathology and the role of astrocytes in a human stem cell model of VCP-related ALS. Cell Rep. 19, 1739–1749 (2017). 28564594 10.1016/j.celrep.2017.05.024 PMC5464993

[89] A. Giblin, A. J. Cammack, N. Blomberg, S. Anoar, A. Mikheenko, M. Carcolé, M. L. Atilano, A. Hull, D. Shen, X. Wei, R. Coneys, L. Zhou, Y. Mohammed, D. Olivier-Jimenez, L. Y. Wang, K. J. Kinghorn, T. Niccoli, A. N. Coyne, R. van der Kant, T. Lashley, M. Giera, L. Partridge, A. M. Isaacs, Neuronal polyunsaturated fatty acids are protective in ALS/FTD. Nat. Neurosci. 28, 737–747 (2025). 40000803 10.1038/s41593-025-01889-3 PMC11976277

[90] M. Hofweber, S. Hutten, B. Bourgeois, E. Spreitzer, A. Niedner-Boblenz, M. Schifferer, M.-D. Ruepp, M. Simons, D. Niessing, T. Madl, D. Dormann, Phase separation of FUS is suppressed by its nuclear import receptor and arginine methylation. Cell 173, 706–719.e13 (2018). 29677514 10.1016/j.cell.2018.03.004

[91] L. A. Gruijs da Silva, D. Dormann, Sedimentation assays to assess the impact of posttranslational modifications on phase separation of RNA-binding proteins in vitro and in cells. Methods Mol. Biol. 2563, 325–339 (2023). 36227481 10.1007/978-1-0716-2663-4_16

[92] P. A. Ewels, A. Peltzer, S. Fillinger, H. Patel, J. Alneberg, A. Wilm, M. U. Garcia, P. Di Tommaso, S. Nahnsen, The nf-core framework for community-curated bioinformatics pipelines. Nat. Biotechnol. 38, 276–278 (2020). 32055031 10.1038/s41587-020-0439-x

[93] Y. Perez-Riverol, J. Bai, C. Bandla, D. García-Seisdedos, S. Hewapathirana, S. Kamatchinathan, D. J. Kundu, A. Prakash, A. Frericks-Zipper, M. Eisenacher, M. Walzer, S. Wang, A. Brazma, J. A. Vizcaíno, The PRIDE database resources in 2022: A hub for mass spectrometry-based proteomics evidences. Nucleic Acids Res. 50, D543–D552 (2022). 34723319 10.1093/nar/gkab1038 PMC8728295

[94] S. Tyanova, T. Temu, P. Sinitcyn, A. Carlson, M. Y. Hein, T. Geiger, M. Mann, J. Cox, The Perseus computational platform for comprehensive analysis of (prote)omics data. Nat. Methods 13, 731–740 (2016). 27348712 10.1038/nmeth.3901

[95] Y. Xiao, T. H. Hsiao, U. Suresh, H. I. Chen, X. Wu, S. E. Wolf, Y. Chen, A novel significance score for gene selection and ranking. Bioinformatics 30, 801–807 (2014). 22321699 10.1093/bioinformatics/btr671 PMC3957066

