# Supplementary material for: An alternative cytoplasmic SFPQ isoform with reduced phase separation potential is up-regulated in ALS
Source: Sci Adv. 2025 Aug 22;11(34):eadt4814. doi: 10.1126/sciadv.adt4814 (PMC12372870; doi:10.1126/sciadv.adt4814)
Supplement: Supplementary file 1 — Figs. S1 to S8 Legends for tables S1 and S2 Tables S3 to S5 [file sciadv.adt4814_sm.pdf]

Science Advances
AAAS

Supplementary Materials for

An alternative cytoplasmic SFPQ isoform with reduced phase separation potential is up-regulated in ALS

Jacob Neeves *et al.*

Corresponding author: Jacob Neeves, jacob.neeves.17@ucl.ac.uk;
Rickie Patani, rickie.patani@ucl.ac.uk, rickie.patani@nus.edu.sg

Sci. Adv. **11**, eadt4814 (2025)
DOI: 10.1126/sciadv.adt4814

The PDF file includes:

Figs. S1 to S8
Legends for tables S1 and S2
Tables S3 to S5

Other Supplementary Material for this manuscript includes the following:

Tables S1 and S2

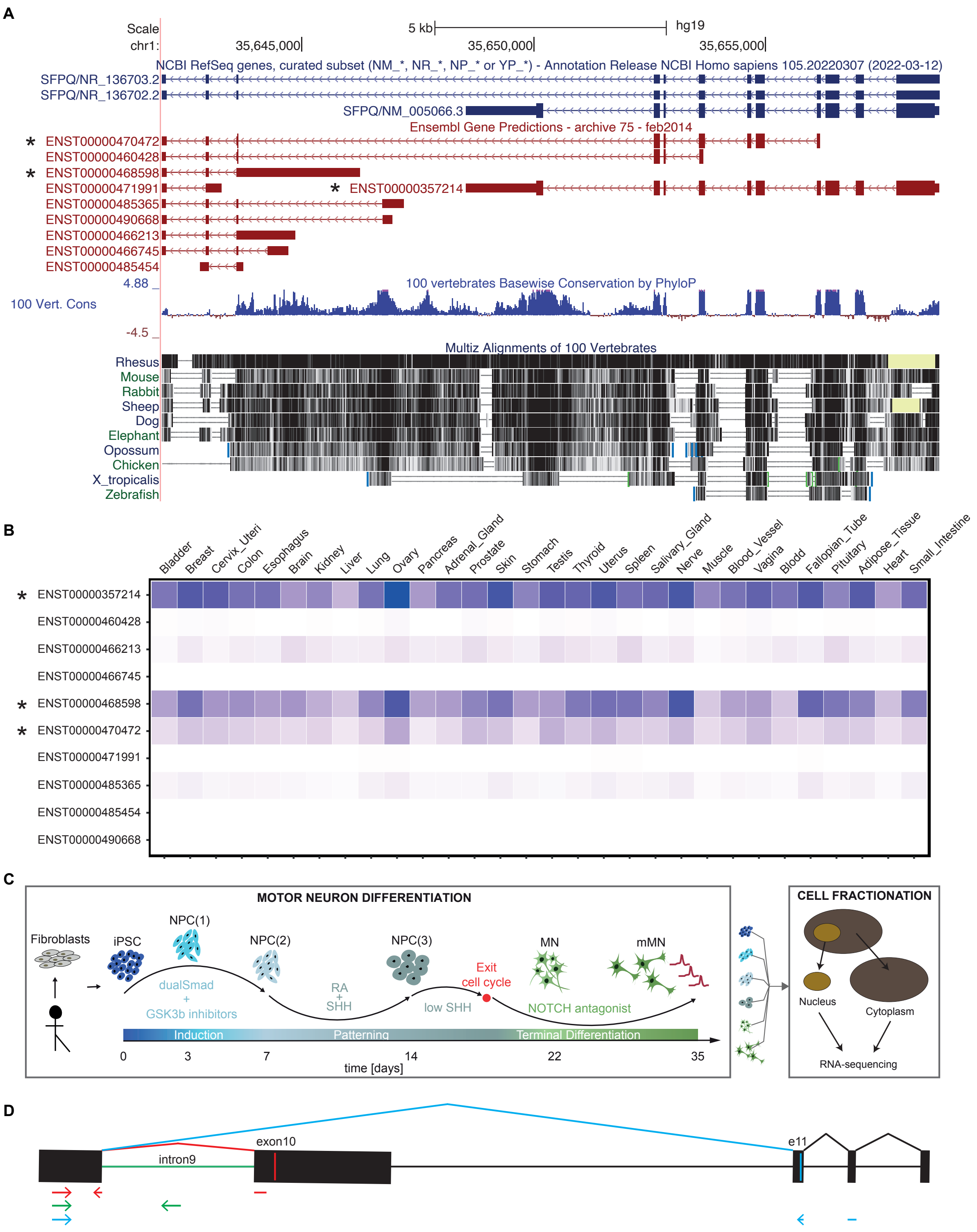

Figure S1: *SFPQ* alternative distal exon splicing is ubiquitous and well conserved. (**A**) UCSC genome browser output for *SFPQ* gene including NCBI RefSeq and all annotated Ensembl transcript isoforms (upper panel); phyloP conservation scores and 100 vertebrate MULTIZ alignments (lower panel). Ensembl 2014 version is displayed to integrate precisely with Figure S1B; the latest version includes just one additional transcript (ENST00000696553), containing the same sequence as ENST00000357214 with an

extended 5'UTR. **(B)** Heatmap generated by ExonSkipDB (https://ccsm.uth.edu/ExonSkipDB/; (*27*)) depicting relative *SFPQ* transcript expression across 31 normal tissues from Genotype-Tissue Expression (GTEx); 3 ubiquitously expressed transcripts are denoted by asterisks in both **A** and **B**. (**C**) Schematic depiction of the hiPSC differentiation strategy for motor neurogenesis adapted from Tyzack et al., (*25*). Sampling time points, in days, are indicated when cells were fractionated into nuclear and cytoplasmic compartments prior to deep [poly(A)] RNA sequencing. Four hiPSC clones were obtained from four different healthy controls. NPCs = neural precursors; MNs = post-mitotic but electrophysiologically inactive motor neurons; mMNs = electrophysiologically active motor neurons. (**D**) SFPQ gene schema displaying the three splicing events of interest all diverging from exon 9 to: exon 10 (red line), exon 11 (blue line), and retention of intron 9 (green bar); vertical-coloured lines denote STOP codons. Primer design strategy represented by coloured arrows.

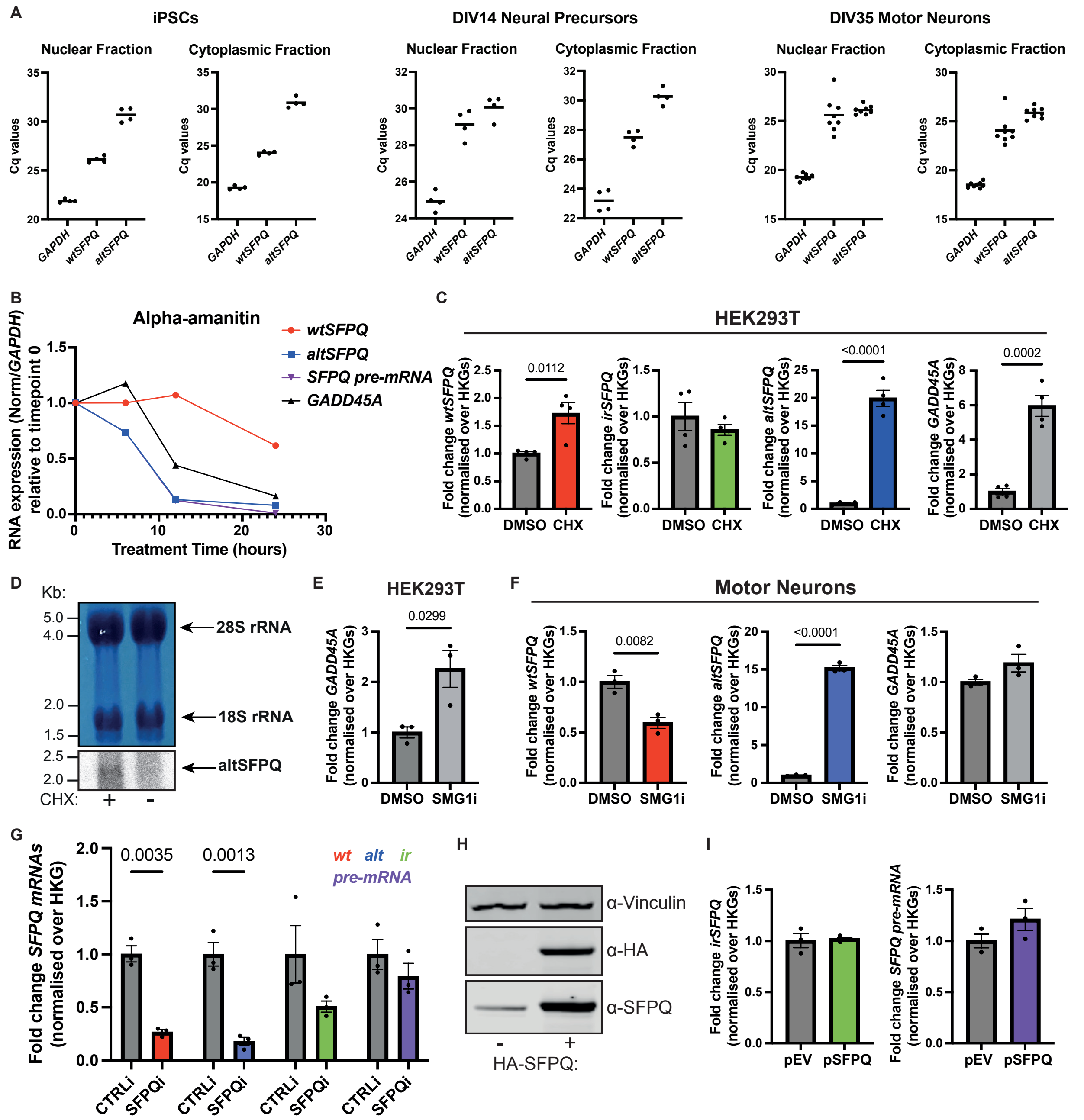

Figure S2: *altSFPQ* is a nonsense-mediated mRNA decay target and contributes to SFPQ autoregulation. (**A**) Cq values for *GAPDH*, *wtSFPQ* and *altSFPQ* as measured by qPCR on nuclear and cytoplasmic fraction samples derived from iPSC, DIV14 neural precursor and DIV35 motor neuronal stages; note that absolute values are comparable within but not between stages, due to different input RNA / cDNA amounts used for RT-qPCR of different stages. (**B**) Line graph displays RNA expression relative to timepoint 0 when irreversible RNA polymerase II inhibitor alpha-amanitin treatment was administered, normalised at each timepoint over *GAPDH* mRNA, in HEK293T cells (n = 1). (**C**) Relative expression levels of transcripts normalised over *GAPDH* & *POLR2B* housekeeping genes, in cycloheximide (CHX) and mock treated HEK293T cells (n = 3; data represents mean +/- SEM; unpaired t tests). (**D**) Northern blot analysis of *altSFPQ* mRNA expression utilising a probe specifically targeting the alt isoform on RNA samples

matched to **Figure S2B** confirms CHX-mediated *altSFPQ* mRNA upregulation; 28S and 18S ribosomal RNAs, detected with methylene blue staining, demonstrate equal sample loading. (**E**) Bar graph showing *GADD45A* (canonical NMD target) mRNA relative expression normalised over *GAPDH* & *POLR2B* housekeeping genes as measured by qPCR, in SMG1 inhibitor (SMG1i) and mock treated HEK293T cells (n = 3; data represents mean +/- SEM; unpaired t test). (**F**) Bar graph showing *wtSFPQ*, *altSFPQ* and *GADD45A* mRNA expression levels normalised over *GAPDH* & *POLR2B* housekeeping genes as measured by qPCR, in SMG1 inhibitor (SMG1i) and mock treated hiPSC-derived day 6 motor neurons (n = 3 control lines; data represents mean +/- SEM; unpaired t tests). (**G**) SFPQ transcript (*wt = red*, *alt = blue*, *ir = green*, *pre-mRNA = purple*) levels normalised over *GAPDH* housekeeping gene as measured by qPCR in SFPQ siRNA treated hiPSC-derived day 3 motor neurons (n = 3; data represents mean +/- SEM; 2-way ANOVA with Sidak's multiple comparisons test). (**H**) N-terminal HA-tagged SFPQ plasmid-derived protein expression assessed by Western blot demonstrating correct expression of an approximate 100 kDa HA-SFPQ. (**I**) Bar graphs showing *irSFPQ & SFPQ pre-mRNA* expression levels normalised over *GAPDH* & *POLR2B* housekeeping genes as measured by qPCR, in HA-wtSFPQ plasmid (pSFPQ) and control plasmid (pEV) transfected HeLa cells (n = 3; data represents mean +/- SEM). HKGs = housekeeping genes.

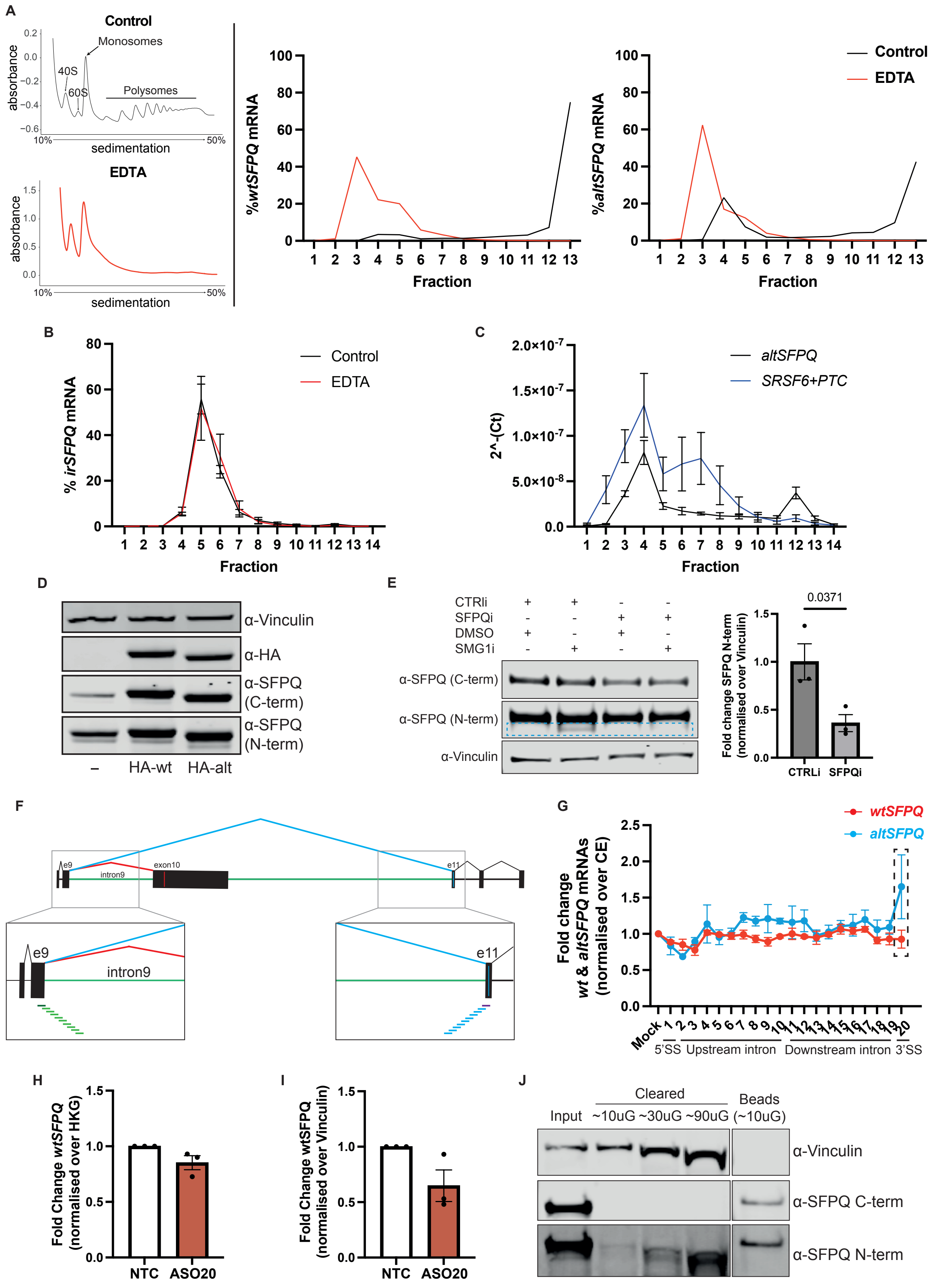
A
Control
Monosomes
40S
60S
Polysomes
absorbance
sedimentation
10%
50%
EDTA
%wtSFPQ mRNA
%altSFPQ mRNA
Fraction
Control
EDTA
B
% irSFPQ mRNA
Fraction
C
2^-(Ct)
altSFPQ
SRSF6+PTC
Fraction
D
α-Vinculin
α-HA
α-SFPQ (C-term)
α-SFPQ (N-term)
–
HA-wt
HA-alt
E
CTRLi
SFPQi
DMSO
SMG1i
α-SFPQ (C-term)
α-SFPQ (N-term)
α-Vinculin
0.0371
Fold change SFPQ N-term (normalised over Vinculin)
CTRLi
SFPQi
F
e9
intron9
exon10
e11
G
wtSFPQ
altSFPQ
Fold change wt & altSFPQ mRNAs (normalised over CE)
Mock
5'SS
Upstream intron
Downstream intron
3'SS
H
Fold Change wtSFPQ (normalised over HKG)
NTC
ASO20
I
Fold Change wtSFPQ (normalised over Vinculin)
NTC
ASO20
J
Input
Cleared
~10uG
~30uG
~90uG
Beads (~10uG)
α-Vinculin
α-SFPQ C-term
α-SFPQ N-term

Figure S3. *AltSFPQ* encodes a novel SFPQ protein. (**A**) Polysome sucrose gradient (10 – 50%) absorbance (A254 nm) profiles obtained from HEK293T cells with (red) or without (black) EDTA treatment of cell lysates to disrupt ribosomes (left panels). EDTA treatment caused substantial loss of polysomes. Targeted analysis (right panels) of *wtSFPQ* or *altSFPQ* mRNA expression by qPCR from each fraction, plotted as percentage of total recovered mRNA from all fractions, in EDTA-treated and untreated cultures (N = 1). (**B**) Targeted analysis of *irSFPQ* mRNA expression by qPCR from each HEK293T fraction, plotted as percentage of total recovered mRNA from all fractions, in EDTA-treated and untreated hiPSC-derived neural precursors (n = 3). (**C**) Head-to-head comparison of abundance of altSFPQ and the NMD-sensitive SRSF6 PTC+ mRNAs measured by qPCR (displayed as 2^-(Ct)) from the polysome profile of hiPSC-derived neural precursors (n = 3). (**D**) Western blot of untreated HEK293Ts or transiently transfected with HA-tagged recombinant SFPQ-expressing plasmids, demonstrating the similar sizes of wtSFPQ and altSFPQ proteins. (**E**) Western blot of control (CTRLi) or SFPQ (SFPQi) siRNA pretreated, SMG1i treated and untreated HEK293T cells, alongside quantification of the altSFPQ protein band, demarcated by the dashed blue box, between control and SFPQ siRNA pretreated SMG1i (+) conditions (n = 3; unpaired t-test). (**F**) Schematic depiction of splicing modulation strategy using overlapping 18 nucleotide PS-2'MOE ASOs targeting 5' and 3' splice sites at either side of the altSFPQ splice junction (labelled as exon 9 and exon 11), and 18 ASOs walked along the intron from 5' or 3' splice site positions. (**G**) Graph depicting fold change over no-ASO mock control of percentage splicing in HEK293Ts for wtSFPQ or altSFPQ (transcripts normalised over constitutive region expression level) determined by qPCR. ASO number corresponds to schematic in **F**. ASO20 highlighted by a dashed box. Data presented as mean +/- SEM from n = 3. (**H**) *WtSFPQ* mRNA levels (normalised over *GAPDH* housekeeping gene) in ASO20 treated HEK293T samples as fold change over non-targeting ASO (NTC) samples, as determined by qPCR (n = 3; data represents mean +/- SEM). (**I**) Densitometry quantification of C-term (ab177149) SFPQ protein bands from samples as described in **2F** & **2G,** using inputs for quantification within each replicate. Displayed as wtSFPQ levels (protein bands normalised over vinculin housekeeping protein) in ASO20 treated HEK293T samples as fold change over non-targeting ASO (NTC) samples. (**J**) Western blot assessment of endogenous SFPQ isoform expression in iPSC-derived motor neurons; samples subjected to IP using C-terminal ab177149 SFPQ antibody. CE = constitutive exon; HKG = housekeeping gene.

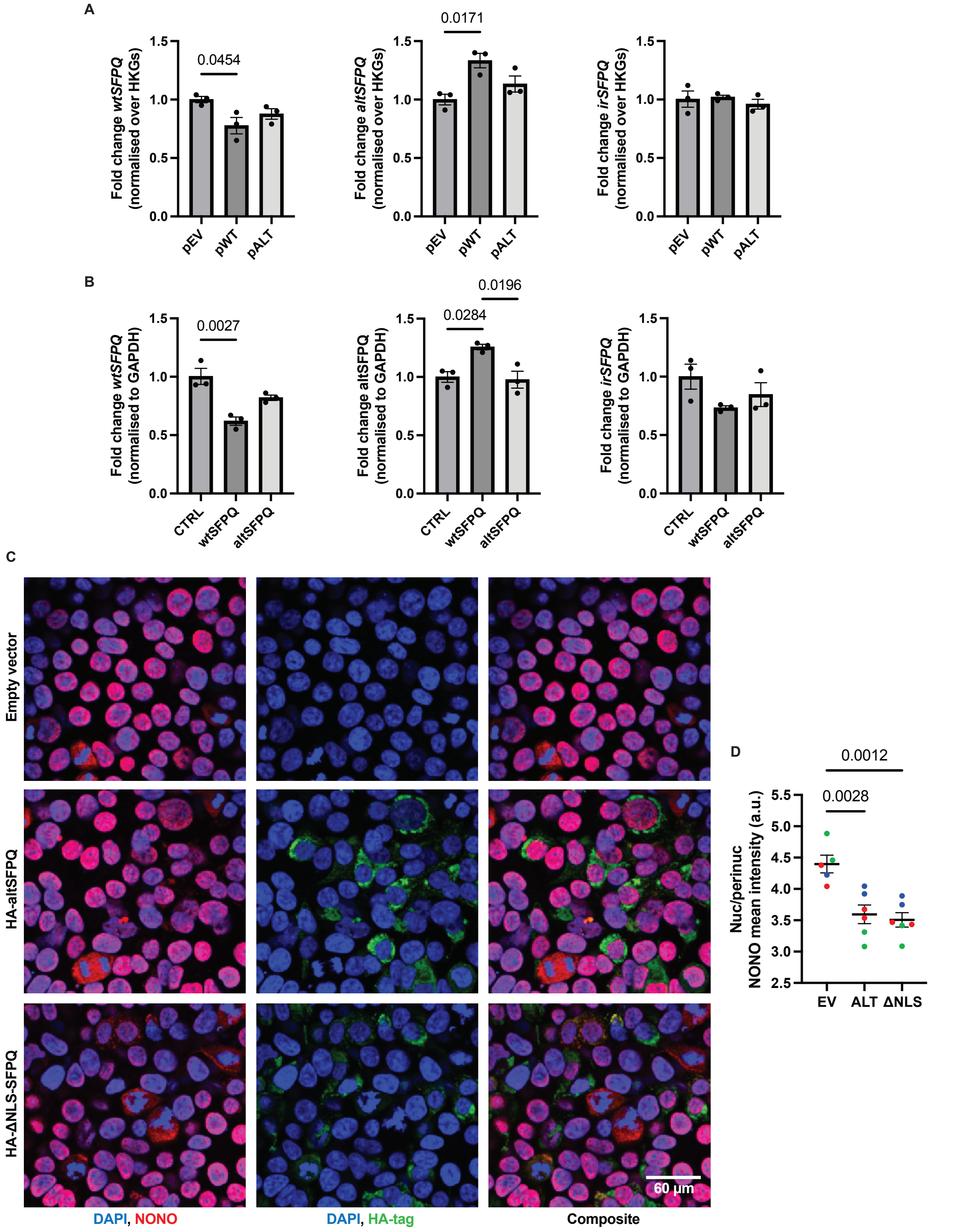

Figure S4. ***AltSFPQ*** **encodes a cytoplasm-predominant protein, which attenuates subcellular distribution of DBHS proteins.** (**A**) Graphs showing *wtSFPQ*, *altSFPQ* and *irSFPQ* expression levels

normalised over *GAPDH* & *POLR2B* housekeeping genes, as measured by qPCR, in HA-wtSFPQ, HA-altSFPQ and empty vector (EV) control plasmid transfected conditions (n = 3; 1-way ANOVA, Tukey's multiple comparisons). (**B**) Graphs showing *wtSFPQ*, *altSFPQ* and *irSFPQ* expression levels normalised over *GAPDH* housekeeping gene, as measured by qPCR, in wtSFPQ-T2A-mAPPLE, altSFPQ-T2A-mAPPLE and mAPPLE control lentiviral transduced DIV14 (day 11 post-transduction) i3Neurons (n = 3; 1-way ANOVA, Tukey's multiple comparisons). (**C**) Representative images of HEK293T cultures transfected with an empty control, HA-altSFPQ, or HA-ΔNLS-wtSFPQ plasmid; immunofluorescence performed using antibodies against HA-tag and NONO and stained for NONO endogenous protein. (**D**) Image analysis quantification relating to **C** measuring nuclear-cytoplasmic ratio (using a 10 um perinuclear ring region) of NONO antibody signal in HEK293T cultures transfected with an empty control, HA-altSFPQ, or ΔNLS-wtSFPQ plasmid (n = 3; each data point represents the average of 36 fields of view per replicate; coloured data points reflect technical replicates; 1-way ANOVA, Tukey's multiple comparisons).

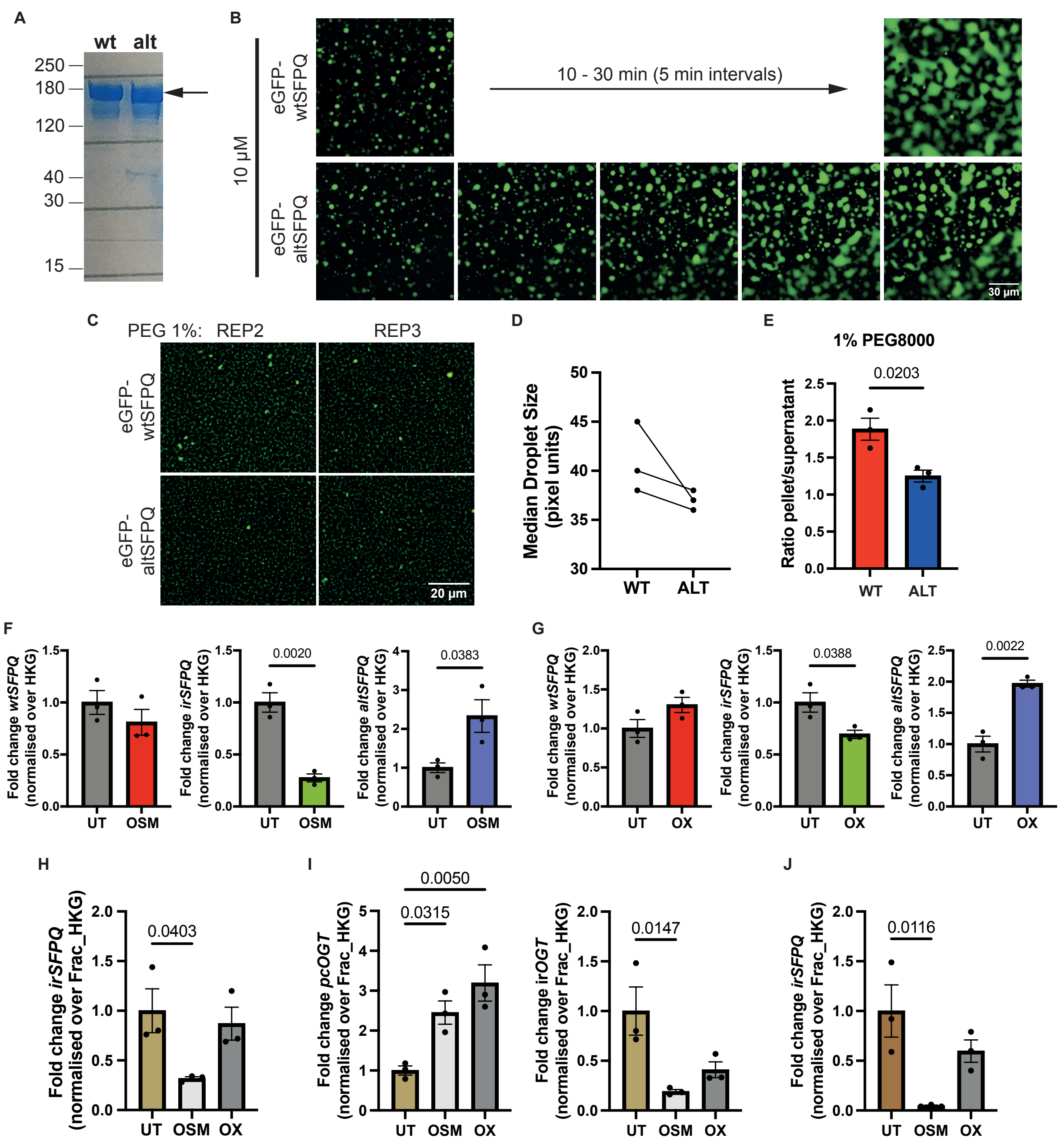

Figure S5. AltSFPQ exhibits reduced phase separation propensity compared to wtSFPQ and is upregulated during neuronal stress. (**A**) Coomassie stained products of tandem affinity protein purification; the arrow indicates purified full length ~180 kDa hisx6-eGFP-SFPQ-MBP proteins. (**B**) eGFP-SFPQ *in vitro* droplet formation following TEV cleavage and addition of 3% PEG crowding agent, using 10 µM protein. PEG was added after 60 min TEV cleavage and images were taken from 10 min after addition of PEG; firstly an image of wtSFPQ was taken, then 5 images of the same field of altSFPQ at 5 min intervals (10 - 30 min after PEG addition), then another image of wtSFPQ at 30 min post-PEG addition. (**C**) LLPS droplet assay representative images demonstrating eGFP-SFPQ protein homotypic liquid droplet formation in 1% PEG crowding agent in two additional experimental replicates. (**D**) Graph showing LLPS droplet sizes for eGFP-tagged wt and alt SFPQ proteins; each data point represents the median droplet area in pixel

units per experimental batch (3 µM protein in 150 mM salt buffer and 1% PEG8000 crowding agent; n = 3). (**E**) Densitometry quantification of sedimented eGFP-wtSFPQ and eGFP-altSFPQ recombinant protein samples processed with the addition of PEG8000 1% crowding agent (n = 3; unpaired t-test). (**F**) Bar graphs displaying expression of *SFPQ* transcripts (normalised over housekeeping gene *GAPDH*) in untreated (UT) and sorbitol-induced osmotically stressed (OSM) day 6 iPSC-motor neurons. Data are expressed as fold change over UT mean +/- SEM from three control lines; unpaired t tests. (**G**) as for **F** but comparing UT and sodium arsenite-induced oxidative stress (OX) conditions. (**H**) Bar graph displaying expression of *irSFPQ* in nuclear fractions from day 6 motor neurons in response to sorbitol-induced osmotic (OSM) and sodium arsenite-induced (OX) stress conditions, normalised over *Nit1* and *NFX1* fraction housekeeping genes. Data are expressed as fold change over untreated (UT) samples per line and presented as mean +/- SEM from three control lines; ANOVA with Dunnett's multiple comparisons test. Frac_HKGs = fraction housekeeping genes. (**I**) Bar graphs displaying expression of spliced protein coding (*pcOGT*) and intron retaining (*irOGT*) *OGT* mRNAs in nuclear fractions from day 6 iPSC-motor neurons in response to sorbitol-induced osmotic (OSM) and sodium arsenite-induced (OX) stress conditions, normalised over *Nit1* and *NFX1* fraction housekeeping genes. Data are expressed as fold change over untreated (UT) samples per line and presented as mean +/- SEM from three control lines; ANOVA with Dunnett's multiple comparisons test. (**J**) Bar graph displaying expression of *irSFPQ* in cytoplasmic fractions from day 6 motor neurons in response to sorbitol-induced osmotic (OSM) and sodium arsenite-induced (OX) stress conditions, normalised over *Nit1* and *NFX1* fraction housekeeping genes. Data are expressed as fold change over untreated (UT) samples per line and presented as mean +/- SEM from three control lines; ANOVA with Dunnett's multiple comparisons test. Frac_HKGs = fraction housekeeping genes.

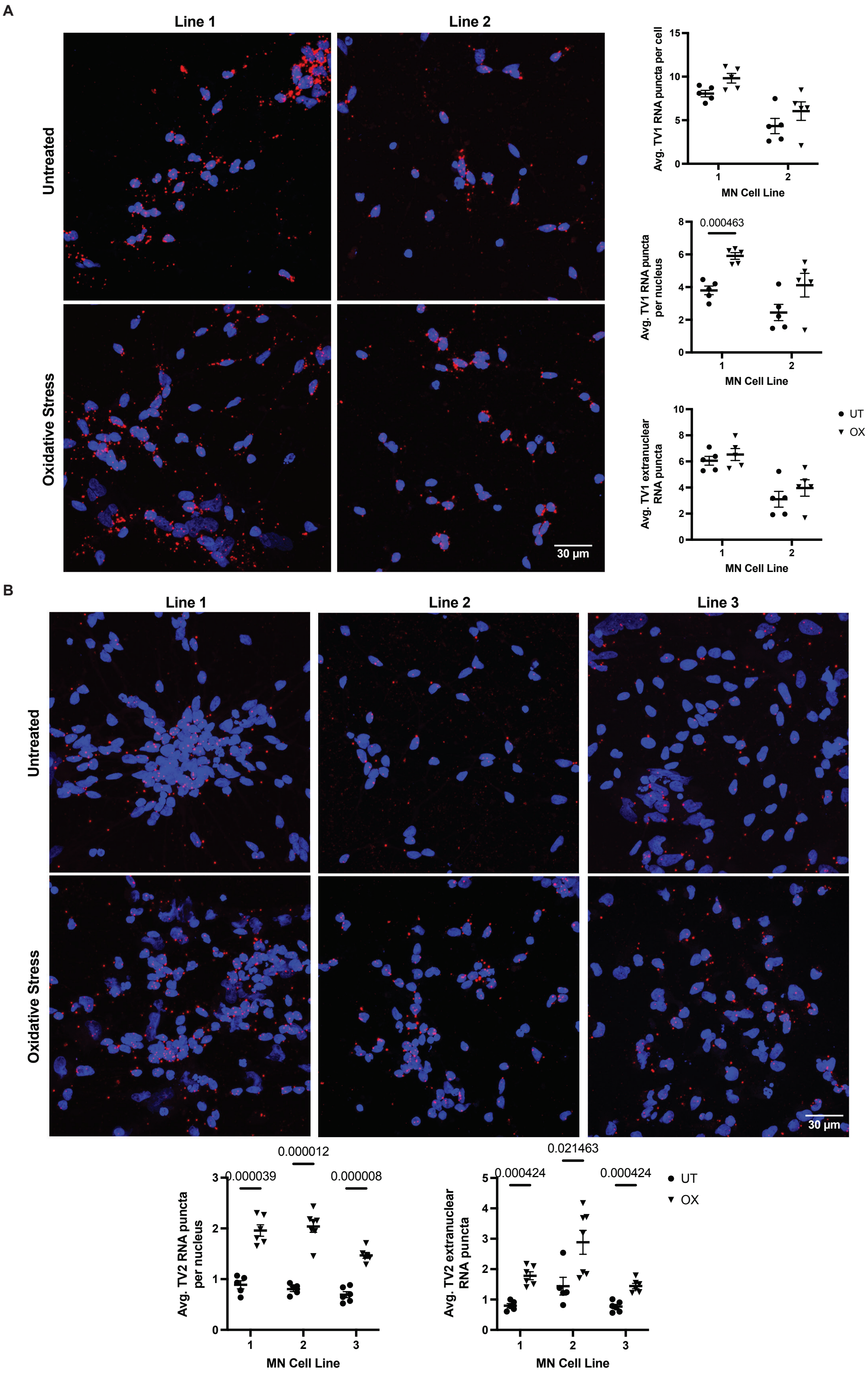
A
Line 1
Line 2
Untreated
Oxidative Stress
30 µm
Avg. TV1 RNA puncta per cell
MN Cell Line
Avg. TV1 RNA puncta per nucleus
0.000463
Avg. TV1 extranuclear RNA puncta
UT
OX
B
Line 1
Line 2
Line 3
Untreated
Oxidative Stress
30 µm
Avg. TV2 RNA puncta per nucleus
0.000039
0.000012
0.000008
MN Cell Line
Avg. TV2 extranuclear RNA puncta
0.000424
0.021463
0.000424
MN Cell Line
UT
OX

Figure S6. BaseScope analyses of oxidative stress-induced *altSFPQ* expression in motor neurons. (**A**) Representative images of BaseScope RNA-FISH on untreated and sodium arsenite (0.5 mM) treated day 6 terminally differentiated motor neurons, probed for *wtSFPQ* (TV1) unique exon junction using 1xZZ probe pairs, alongside quantification of average RNA puncta per cell, nucleus, or extranuclear cell region in two control lines. (**B**) Confocal images of BaseScope RNA-FISH on untreated and sodium arsenite (0.5 mM) treated day 6 terminally differentiated motor neurons, probed for *altSFPQ* (TV2) unique exon junction using 1xZZ probe pairs, alongside quantification of average RNA puncta per cell, nucleus, or extranuclear cell region in three control lines. Data points reflect individual imaged fields of view per biological line; multiple unpaired t-tests. UT = untreated; OX = sodium-arsenite oxidative stress.

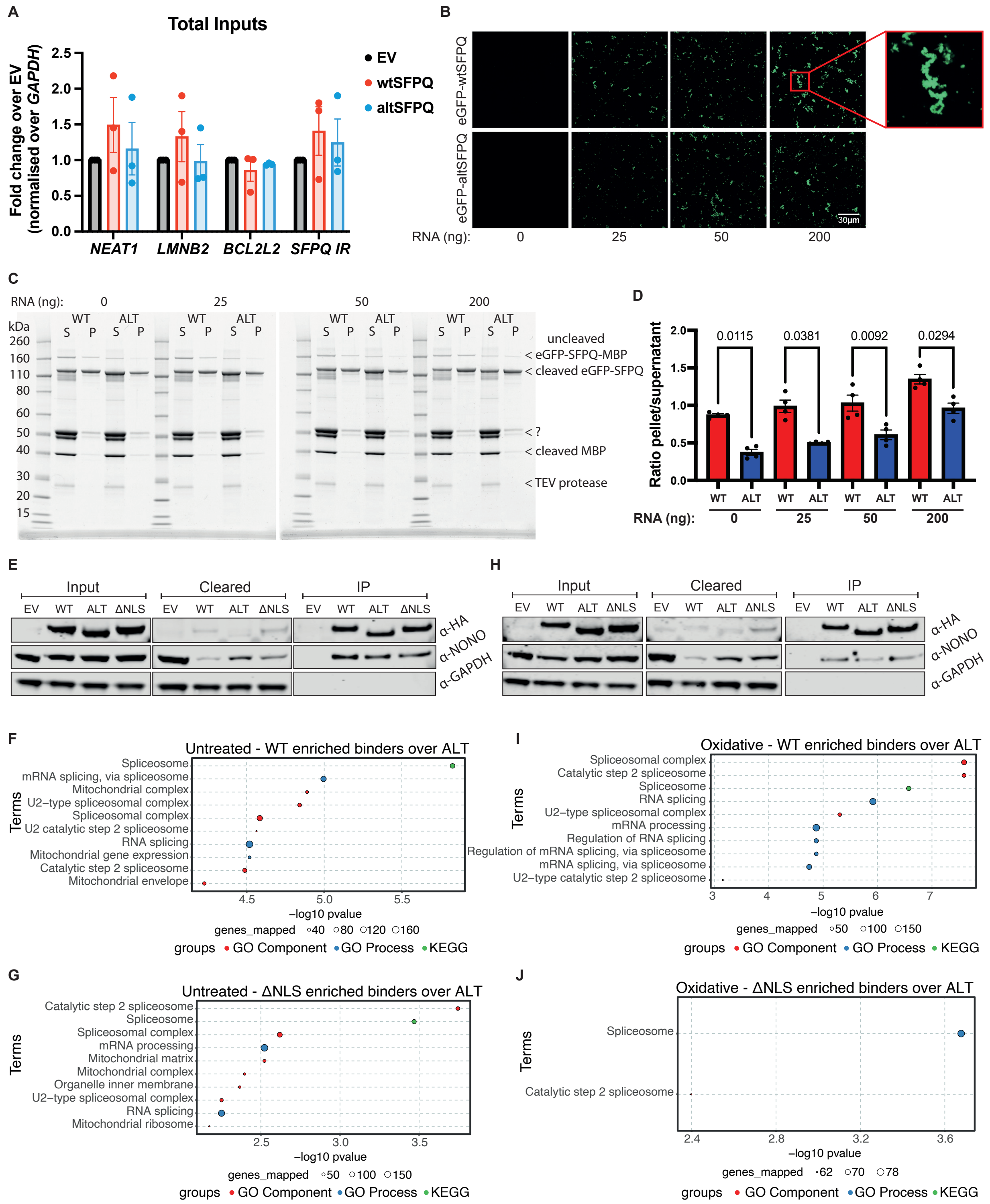

Figure S7. Altered C-terminus drives differential protein binding partners for altSFPQ. (**A**) Bar graphs showing expression of indicated transcripts in total inputs from N2A cells expressing HA-SFPQ variants or empty vector (EV). Values, derived from qPCR analysis, were normalised to *GAPDH* housekeeping gene, then expressed as fold change over empty vector control within each replicate (n = 3). (**B**) Confocal images of eGFP-SFPQ proteins with increasing amounts of an 800 bp sequence of *SFPQ intron 9* RNA added.

Zoomed inset displays typical protein structure formed by the proteins, presumably bound to the RNA. (**C**) Representative image of sedimentation assay gel loaded with supernatant and pellet fractions of TEV cleaved eGFP-wtSFPQ and eGFP-altSFPQ recombinant proteins with increasing amounts of an 800 bp sequence of *SFPQ intron 9* RNA added. (**D**) Densitometry quantification of the ratio of cleaved eGFP-SFPQ proteins in pellet / supernatant fractions, for TEV cleaved samples with increasing amounts of *SFPQ intron 9* RNA added (n = 3; multiple unpaired t-tests). (**E**) Western blot showing HA-tagged SFPQ protein variants and co-immunoprecipitated NONO binding partner but not GAPDH, using anti-HA, -NONO, and -GAPDH antibodies, in untreated samples. (**F**) ORA of 808 SFPQ-bound proteins (subsetted based on > 0.3 positive correlation with the bait HA-proteins) comparing WT vs ALT binding enrichment from untreated cells (top 10 GO pathways & KEGG terms displayed). (**G**) As for **F** but for ΔNLS-SFPQ enriched terms over altSFPQ. (**H**) As for **E** but in oxidative-stressed cells. (**I**) As for **F** but in oxidative-stressed cells. (**J**) As for **G** in oxidative-stressed cells. Some GO terms are shortened.

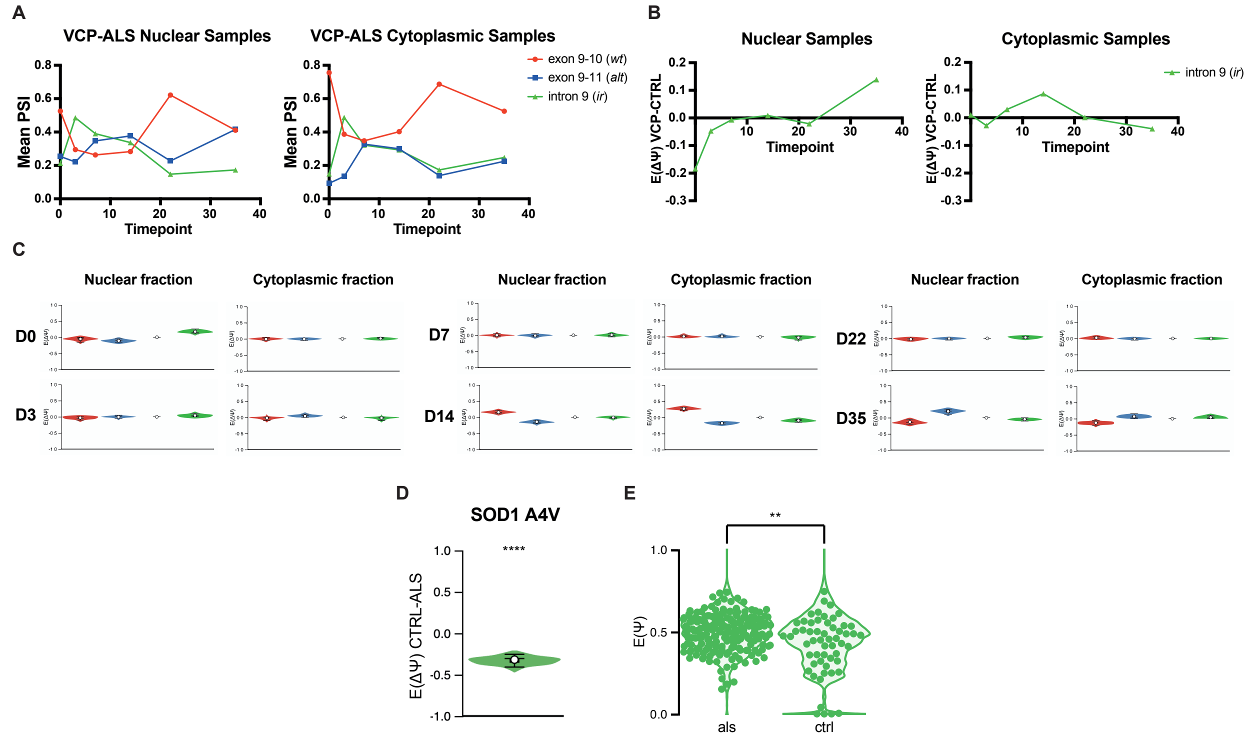

Figure S8: *SFPQ* is dysregulated in familial and sporadic ALS iPSMNs. (**A**) Line graphs show mean PSI for each of the three *SFPQ* splicing events across six stages of neuronal differentiation in either nuclear (left panel) or cytoplasmic (right panel) fractions derived from four VCP ALS patient hiPSC lines. (**B**) Line graph showing mean delta PSI value (*VCP* mutant- Control) for intron 9 splicing across 6 stages of neuronal differentiation in either nuclear (left panel) or cytoplasmic (right panel) fractions derived from four control and four VCP ALS patient hiPSC lines. (**C**) Violin plots showing CTRL-VCP deltaPSI values for all *SFPQ* splicing events within LSV chr1:35187001-35187122 across six stages of neuronal differentiation in either nuclear or cytoplasmic fractions derived from four control and four VCP ALS patient hiPSC lines. A very lowly expressed additional splice junction (white dot) was detected in this analysis, which exhibited no change in VCP-ALS samples. (**D**) Violin plot showing CTRL-SOD1 deltaPSI values (y-axis) for *SFPQ* intron 9 splicing in *SOD1* A4V mutant motor neurons versus controls (Kiskinis et al., (*56*)). (**E**) Violin plots showing PSI values (y-axis) for *SFPQ* intron 9 splicing event in >200 sporadic ALS hiPSC-derived motor neurons and >50 control samples.

Table S1: List of proteins bound by WT-, ALT- and ΔNLS-SFPQ proteins over the empty vector control (log2FC > 0.5, $p < 0.05$) in affinity purification experiments on untreated and oxidative-stressed cells.

Table S2: List of comparative binding of proteins to WT-, ALT- or ΔNLS-SFPQ in affinity purification experiments on untreated and oxidative-stressed cells, as well as π-values (log2FC × -log10 p-value) for the 808 proteins with positive correlation to hSFPQ (Pearson > 0.3) used for ORA.

iPSC cell line name	Mutation	Age of the donor	Age at disease onset	Sex of the donor	Comments
CTRL1	None	78	n/a	Male	
CTRL2	None	64	n/a	Male	
CTRL3	None	unknown	n/a	Female	
CTRL4	None	51	n/a	Female	
CTRL5	None	51	n/a	Male	
MUT1	R155C	43	40	Female	Clone from same patient as MUT2
MUT2	R155C	43	40	Female	Clone from same patient as MUT1
MUT3	R191Q	42	36	Male	Clone from same patient as MUT4
MUT4	R191Q	42	36	Male	Clone from same patient as MUT3

Table S3: Human induced pluripotent cell lines used in study

Target	Forward	Reverse
GAPDH (human)	ATGACATCAAGAAGGTGGTG	CATACCAGGAAATGAGCTTG
POLR2B (human)	CAGAAAAGGTTCTGATTGCCCAAGA	AATGCGCTGACCAATAGCAC
NFX1	GACTGTGGACCATGCTCTCG	TGTAGCATCTTCACTTTTGAGACT
NIT1	GGCTATATCTTCATGCTGGGCT	GGGCTGAGCACAAAGTACTGA
SFPQ Constitutive	ACGGGAAAGAGACATGCGAA	TGGTGGAACGCCAGGATTAG
wtSFPQ	GATGGGAAGTGACATGCGTA	TTCCTCTAGGACCCTGTCCA
altSFPQ	GGTGGTGGTGGCATAGGTTA	GCTGTGTGATGTCTTGAGAAGT
irSFPQ	GTGGATCGACTCATTGGTGA	TTCCTCTAGGACCCTGTCCA
SFPQ pre-mRNA (intron 7)	CAGCCTTTACTGCCTTCCAA	TCCTCTGACCCATACAAGGA
wtSFPQ 3’ UTR	GAAGTGACATGCGTACTGAGC	AACAAACTGGAATGAAAGCCTAAA
OGT Constitutive	CACTTCCAGTGTCGAAGGCT	GCCAGAAGGGGGTTCTGTTT
pcOGT	ATGGAAGGGGCAGTACAAGC	GCAAAGTTCGGTTGCGTCTC
OGT IR Transcript	GAAGCCAAGGTAGGTGTTTGA	AAGAGTTGAAGACTTGGCAAA
FUS IR Transcript	AGCAGTGGTGGCTATGAACC	GCACTAGGGACTGGCTTCAG
GADD45A	GTTTTGCTGCGAGAACGACA	TCCTTCCATTGAGATGAATGTGG
SRSF6 PTC+	GGAAGCCGCATGACCAAT	TAGGGCAAGGGTCACACAAT
GAPDH (mouse)	GAAGGTCGGTGTGAACGGAT	ACTGTGCCGTTGAATTTGCC
POLR2B (mouse)	AGATGTATGACGCCGACGAG	TCACGATCCAGCATGCTTCC
irSFPQ (mouse)	GGTGGTGGTGGCATAGGTTA	TCTCCTTTGTACTATGTCCCACAG
NEAT1 (mouse)	CAGGAGGCCATCGTTGAAGT	CTGCTGCCATTCATGCATCC
LMNB2 (mouse)	CAGGATGTGGTGGCTCTTAGG	CAGAGAAGCCTGTGAATGCC
BCL2L2 (mouse)	TACCTGCCATGACCTAACGC	AGGGGACTGGACTGTGAGTT

Table S4: Primers used in study

Antibody	Species	Application	Cat #
Vinculin	Rabbit	WB	42H89L44, ThermoFisher
Lamin-B1	Rabbit	WB	D4Q4Z, Cell Signaling
SFPQ (C-terminal)	Rabbit	WB	Ab177149, Abcam
SFPQ (N-terminal)	Mouse	WB	6D7, Novus Biologicals
HA-tag	Rabbit	WB / ICC	C29F4, Cell Signaling
GAPDH	Rabbit	WB	AM4300, Ambion
NONO	Rabbit	WB / ICC	Ab70335, Abcam
TIA-1	Goat	ICC	SC-1751, Santa Cruz

Table S5: Antibodies used in study
